# Imidazopyridazine Inhibitors of Plasmodium falciparum Calcium-Dependent Protein Kinase 1 Also Target Cyclic GMP-Dependent Protein Kinase and Heat Shock Protein 90 To Kill the Parasite at Different Stages of Intracellular Development

**DOI:** 10.1128/AAC.01748-15

**Published:** 2016-02-26

**Authors:** Judith L. Green, Robert W. Moon, David Whalley, Paul W. Bowyer, Claire Wallace, Ankit Rochani, Rishi K. Nageshan, Steven A. Howell, Munira Grainger, Hayley M. Jones, Keith H. Ansell, Timothy M. Chapman, Debra L. Taylor, Simon A. Osborne, David A. Baker, Utpal Tatu, Anthony A. Holder

**Affiliations:** a The Francis Crick Institute, Mill Hill Laboratory, London, United Kingdom; b Centre for Therapeutics Discovery, MRC Technology, London, United Kingdom; c Faculty of Infectious and Tropical Diseases, London School of Hygiene and Tropical Medicine, London, United Kingdom; d Department of Biochemistry, Indian Institute of Science, Bangalore, India

## Abstract

Imidazopyridazine compounds are potent, ATP-competitive inhibitors of calcium-dependent protein kinase 1 (CDPK1) and of Plasmodium falciparum parasite growth *in vitro*. Here, we show that these compounds can be divided into two classes depending on the nature of the aromatic linker between the core and the R2 substituent group. Class 1 compounds have a pyrimidine linker and inhibit parasite growth at late schizogony, whereas class 2 compounds have a nonpyrimidine linker and inhibit growth in the trophozoite stage, indicating different modes of action for the two classes. The compounds also inhibited cyclic GMP (cGMP)-dependent protein kinase (PKG), and their potency against this enzyme was greatly reduced by substitution of the enzyme's gatekeeper residue at the ATP binding site. The effectiveness of the class 1 compounds against a parasite line expressing the modified PKG was also substantially reduced, suggesting that these compounds kill the parasite primarily through inhibition of PKG rather than CDPK1. HSP90 was identified as a binding partner of class 2 compounds, and a representative compound bound to the ATP binding site in the N-terminal domain of HSP90. Reducing the size of the gatekeeper residue of CDPK1 enabled inhibition of the enzyme by bumped kinase inhibitors; however, a parasite line expressing the modified enzyme showed no change in sensitivity to these compounds. Taken together, these findings suggest that CDPK1 may not be a suitable target for further inhibitor development and that the primary mechanism through which the imidazopyridazines kill parasites is by inhibition of PKG or HSP90.

## INTRODUCTION

There is a great need to identify novel targets for antimalarial therapeutic intervention (1) since the eventual selection of resistance to drug treatments has repeatedly been a problem in the treatment of malaria (2). This is exemplified by the emergence of Plasmodium falciparum strains displaying delayed clearance by the current treatment of choice, artemisinin and its derivatives, and it is considered inevitable that artemisinin resistance will eventually develop and spread (3–5). Recently mutations in the kelch propeller domain protein, K13-propeller, have been shown to be associated with artemisinin resistance *in vitro* and *in vivo* (6). New drugs, acting on novel targets, are clearly needed in the ongoing fight against malaria. It is for these reasons that Plasmodium protein kinases are now being considered potential drug targets (7, 8).

Calcium-dependent protein kinase 1 (CDPK1) is an abundant protein expressed in many stages of the Plasmodium life cycle. It is indispensable for the development of sexual stages of Plasmodium berghei, playing a role in activating translation of repressed mRNAs (9). In the asexual blood stages, the protein is found at the periphery of merozoites and is associated with the parasite plasma membrane by virtue of myristoylation and palmitoylation of its N terminus (10, 11). Its function in blood stages is elusive, with suggestions that it may play a role in regulating parasite motility. The location of the protein on the inner face of the plasma membrane makes it ideally placed to phosphorylate components of the parasite's motor complex that is anchored to both the parasite plasma membrane (PPM) and the inner membrane complex (IMC). While it has been demonstrated that CDPK1 can phosphorylate the myosin light chain MTIP (myosin A tail domain-interacting protein) and the glideosome-associated protein GAP45 *in vitro* (10) and that these proteins are modified accordingly in the parasite (12–14), there is no direct evidence that CDPK1 is responsible *in vivo*. An early study in P. falciparum suggested that while the *cdpk1* gene was amenable to genetic manipulation, it was not possible to knock the gene out (15). This suggested an essential role for the protein in blood stages. Initial attempts to delete *cdpk1* in P. berghei also proved unsuccessful (16); however, in a more recent study the gene was successfully deleted (17).

Apicomplexan CDPKs have been the subject of several drug development programs; these have been summarized in a recent review article (18). There have been a number of studies describing the development of small-molecule inhibitors of CDPK1. For example, the 2,6,9-trisubstituted purine purfalcamine, which blocks P. falciparum growth, has been shown by affinity purification to bind to CDPK1 (15). Parasites treated with purfalcamine do not progress through asexual blood stage development, arresting when they are very mature schizonts. This inhibition of merozoite egress suggested a possible role for CDPK1 in this process (15). In a second study, two series of small-molecule inhibitors that were competitive for ATP binding to CDPK1 were described (19). We have previously described a series of imidazopyridazine compounds that are potent inhibitors of CDPK1 *in vitro*, with 50% inhibitory concentrations (IC_50_s) in the subnanomolar range (20–23). These inhibitors are ATP competitive; their binding to the recombinant kinase was reduced by a large amino acid at the gatekeeper position and at high concentrations of ATP. Although the most potent of the compounds exhibited a 50% effective concentration (EC_50_) of 12 nM for killing P. falciparum in culture, the compounds performed disappointingly in a rodent model of malaria infection. In addition, one puzzling feature of these inhibitors was the poor correlation between their ability to inhibit the enzyme and their ability to block parasite growth, which was suggestive of off-target activity contributing to their inhibitory effects (23).

Here, we examine the mechanism of action of some of the inhibitors described previously and show that they fall into two classes, causing parasite death at two distinct points of the asexual blood stage cycle. We have identified the likely additional target proteins of each class of compound and have been able to identify features of the compounds that confer this selectivity. Furthermore, using a chemical genetics approach, we show that inhibition of CDPK1 does not appear to affect blood stage parasite growth or survival, leading us to conclude that CDPK1 may not be a suitable target for pharmaceutical intervention for the treatment of blood stage malaria infection.

## MATERIALS AND METHODS

### Parasite lines and culture methods.

All P. falciparum lines were maintained in human erythrocytes provided by the National Blood Transfusion Service. 3D7 is a cloned line obtained from the University of Edinburgh. The 3D7 cyclic GMP (cGMP)-dependent protein kinase (PKG) with a T618Q substitution has been described previously (24). Parasites were grown at 2% hematocrit in RPMI 1640 medium supplemented with 1% Albumax according to published methods (25). Synchronization was achieved by centrifugation through a Percoll gradient (26).

### Drug treatment and SYBR green assay.

Aliquots of 100 μl of P. falciparum cultures 24 h after erythrocyte invasion were transferred into 96-well culture dishes. Cells were incubated with inhibitors for 48 or 96 h (starting parasitemias were 0.3% and 0.03%, respectively). All drug treatments were carried out in duplicate at a final dimethyl sulfoxide (DMSO) concentration of 0.05%. After incubation, a modification of a previously described SYBR green assay was performed (27). Cells were lysed by the addition of 25 μl of buffer (20 mM Tris-HCl, pH 8.0, 2 mM EDTA, pH 8.0, 1.6% Triton X-100, 0.16% saponin, 10× SYBR green I [Life Technologies]). After incubation in the dark for 2 h, fluorescence of the samples was determined using a FLUOStar Omega plate reader (BMG Labtech) with excitation and emission filters of 485 nm and 520 nm, respectively. EC_50_s were calculated from a four-parameter logistical fit of the data using Prism software (GraphPad Software, Inc.). The compounds 1-(1, 1-dimethylethyl)-3-(1-naphthalenyl)-1H-pyrazolo[3,4-d]pyrimidin-4-amine (NA-PP1) and 1-(1, 1-dimethylethyl)-3-(1-naphthalenylmethyl)-1H-pyrazolo[3,4-d]pyrimidin-4-amine (NM-PP1) were obtained from Merck.

### Generation and screening of parasites expressing gatekeeper mutant CDPK1.

Parasites expressing CDPK1 T145G and CDPK1 T145T were generated as described previously (28). Briefly, a region of homology to facilitate integration of the plasmid via single-crossover homologous recombination was amplified from P. falciparum 3D7 genomic DNA at 194 bp upstream of the ATG to bp 435 of the open reading frame using primers 1 and 2 for the wild-type (WT) version and primers 1 and 3 for the glycine version. Each of these fragments was cloned via XmaI and EcoRI sites into a Geneart vector containing a recodonized gene fragment from bp 436 to 1572 of the P. falciparum *cdpk1* (*Pfcdpk1*) open reading frame. Together, the native and recodonized *cdpk1* sequences were cloned between XmaI and AvrII sites of the pHH4-HA plasmid (29), which adds a triple hemagglutinin (HA) tag at the 3′ end, followed by a stop codon and the PbDT-3′ UTR (the 3′ untranslated region of the P. berghei dihydrofolate reductase [DHFR]-thymidylate synthase) and human DHFR (hDHFR) drug selection cassette. Primers were the following: primer 1, ACACCCCGGGGTATACAACGTATAAGACAAATTACTTTTCTTTC; primer 2, ATATGAATTCGGTTACTAAATAAAAATATTTCTTATCTTCAAAAACATCAAAC; primer 3, ATATGAATTCGCCTACTAAATAAAAATATTTCTTATCTTCAAAAACATCAAAC.

P. falciparum 3D7 parasites were transfected with pHH4-CDPK1-HA plasmids (T145T or T145G) using standard methods (30, 31). They were maintained under drug pressure (25 nM WR99210) until resistant parasites emerged, cycled on/off drug, and cloned by limiting dilution.

Clones were screened by PCR using primers 4 and 5 to detect the intact locus and primers 4 and 6 to confirm integration, as well as by Western blotting and immunofluorescence using anti-CDPK1 and anti-HA antibodies. The following are the primer sequences: primer 4, GATGGTGGCACTTGCCTTTTTGAGG; primer 5, CTGGTTTAATATCTCGATGTACAATATTATGTTTATG; primer 6, CCCAATCTGTCCCTTAGCTTGTTGTC.

### Western blotting and immunofluorescence.

Late-schizont-stage parasites were lysed in 1% NP-40, 150 mM NaCl, and 10 mM Tris-HCl, pH 8.0, containing 1× Complete protease inhibitors (Roche). After centrifugation for 20 min at 15,000 × *g*, 10 μg of soluble protein was separated on a 10% Bis-Tris NuPAGE gel (Life Technologies). Transfer to a nitrocellulose membrane was performed using an iBlot system (Life Technologies). Antibodies against HA (rat monoclonal antibody 3F10; Roche) and CDPK1 (10) were used to detect modified or total CDPK1, respectively, using standard methods.

### Recombinant protein production.

PfCDPK1 and PfCDPK1 T145G proteins were produced using methods described elsewhere (23). The PfHSP90 N-terminal domain (NTD) comprises the first 223 amino acids of PfHSP90. This region was amplified from the full-length PfHSP90-pRSET-A construct (32) using the following primers: primer 7, GGCGACGGATCCATGTCAACGGAAACATTCGC; primer 8, GACCCCCTCGAGCTATTCTTCTTCAGATGCGG.

The PCR product was cloned into pRSET-A (Life Technologies) between the BamHI and XhoI sites. Positive clones were confirmed by restriction digestion and sequencing. The clone was transformed into Escherichia coli Rosetta(pLysS) cells, and protein was expressed by induction with 0.1 mM isopropyl β-d-1-thiogalactopyranoside (IPTG) at 37°C for 2 h. PfHSP90 proteins were purified using Ni-nitrilotriacetic acid (NTA) affinity chromatography (Qiagen) as described in the manufacturer's protocol.

### ParM ADP biosensor assay.

IC_50_s were determined in kinetic mode using a rhodamine-labeled ParM (Rh-ParM) ADP sensor (33). For these experiments 5 μl of PfCDPK1 (WT or T145G) was diluted in assay buffer (50 mM Tris-HCl, pH 8.0, 200 μM CaCl_2_, 1 mM dithiothreitol [DTT], 25 mM KCl, 100 μM EGTA, and 0.01% [vol/vol] Triton X-100) to a final concentration of 100 nM and mixed with 10 μl of Rh-ParM at a final concentration of 100 nM in black 384-well plates (Corning). Compounds were diluted in half-log series in DMSO, and 2-μl volumes of diluted compound, or DMSO alone, were added to the enzyme and incubated for 30 min at room temperature. A further control reaction had DMSO but no enzyme. The reaction was initiated with 5 μl of 20 mM MgCl_2_ and ATP at the appropriate previously determined *K_m_* values (23) of 30 μM and 90 μM ATP for WT CDPK1 and CDPK1 T145G, respectively. IC_50_s were calculated from a four-parameter logistical fit of the data using Prism software (GraphPad Software Inc.).

### Expression and purification of recombinant PKG.

Full-length PfPKG and PfPKG T618Q were prepared as described previously (24, 34). Briefly, plasmids (pTrcHisC) encoding full-length PfPKG and PfPKG T618Q with N-terminal His tags were transformed into E. coli Rosetta2(DE3). Single colonies were grown in LB rich broth (supplemented with 50 μg/ml carbenicillin and 34 μg/ml chloramphenicol) at 37°C. Protein expression was induced with 1 mM IPTG once an optical density of 0.6 to 0.7 had been reached. Subsequent overnight growth was at 16°C. PKGs were purified via the histidine tag on HiTrap Talon columns (GE Healthcare) according to the manufacturer's instructions and then concentrated on 10-kDa-molecular-mass-cutoff concentrators (Amicon). Purified proteins were stored in 50% glycerol at −80°C in single-use aliquots. Final buffer composition of the purified product was 50 mM Tris-HCl, pH 7.5, 0.1 mM EGTA, 150 mM NaCl, 0.1% β-mercaptoethanol, 50% glycerol, 0.03% Brij-35, 1 mM benzamidine, and 0.2 mM phenylmethylsulfonyl fluoride (PMSF).

### Assay of cGMP-dependent protein kinase activity.

IC_50_s were determined for test compounds using a microfluidic fluorescent shift assay (unpublished data). Briefly, compounds were prepared over a 10-well half-log dilution series in DMSO in duplicate in 50-μl volumes using 96-well polypropylene U-bottomed plates (Thermo Scientific, United Kingdom). The reaction mix for each well consisted of 20 μl of enzyme/peptide mix (1.25 nM PfPKG or PfPKG T618Q, 1.5 μM 6-carboxyfluorescein [FAM]-labeled PKAtide [FAM-GRTGRRNSI-NH2; Cambridge Bioscience, United Kingdom] in PfPKG assay buffer [25 mM HEPES (pH 7.4), 20 mM β-glycerophosphate, 2 mM DTT, 10 μM cGMP, 0.01% (wt/vol) bovine serum albumin (BSA), 0.01% (vol/vol) Triton X-100]) plus 5 μl of compound. Samples were preincubated at room temperature for 30 min, and reactions were initiated by addition of 25 μl of ATP mix (10 mM MgCl_2_ and ATP, at the *K_m_* of the enzyme under test [20 μM PfPKG and 90 μM PfPKG T618Q], in water). Reactions were terminated at approximately 10% substrate conversion by addition of 50 μl of stop solution (25 mM EDTA in water). Samples were analyzed by electrophoretic separation of substrate and product peak and fluorescence detection using a Caliper LabChip EZ Reader (PerkinElmer, Waltham MA) with a 0.2-s buffer sip time, downstream voltage of 500 V, upstream voltage of 1,950 V, and pressure of 0.5 to 1.5 lb/in^2^. IC_50_s for the compounds were determined using a four-parameter logistical fit of the data (GraphPad Prism).

### Synthesis of biotinylated compound D.

The chemical synthesis of compound D with biotin attached to the R1 or R2 group is described in Information S1 in the supplemental material.

### Affinity purification of targets of compound D.

A total of 200 μl of high-capacity streptavidin agarose (Thermo Scientific) was resuspended in 1 ml of 250 μg/ml biotinylated compound D solution or a DMSO control. After incubation with mixing for 1 h at room temperature, unbound compound was removed by extensive washing with phosphate-buffered saline (PBS). The resin was incubated with trophozoite lysate containing 2 mg of protein in a total volume of 1.5 ml overnight at 4°C. The lysate was prepared by resuspending trophozoites in 20 mM Tris-HCl, pH 8.0, 10 mM MgCl_2_, 250 mM NaCl, 0.5 mM tris(2-carboxyethyl)phosphine (TCEP), 1× protein phosphatase inhibitor (Sigma-Aldrich), 1× complete protease inhibitors (Roche), and 0.1% Triton X-100 (Sigma-Aldrich). Insoluble proteins were removed by centrifugation at 15,000 × *g* for 20 min, and protein was quantitated using a detergent-compatible (DC) protein assay (Bio-Rad). The resin was extensively washed in lysis buffer to remove unbound proteins, and proteins were eluted by resuspending the resin in 100 μl of 2× reducing lithium dodecyl sulfate (LDS) sample buffer (Life Technologies) and heated at 95°C for 5 min.

Proteins were run 4 mm into a 10% NuPAGE Bis-Tris gel (Life Technologies) and then excised using a clean scalpel blade. Proteins were reduced and alkylated prior to overnight trypsin digestion. The resulting digests were analyzed by liquid chromatography-tandem mass spectrometry (LC-MS/MS) using an Ultimate 3000 nanoRSLC high-performance liquid chromatograph (HPLC), equipped with a 50-cm by 75-μm Acclaim Pepmap C_18_ column, coupled to a linear trap quadrupole (LTQ) Orbitrap Velos Pro equipped with a Nanoflex electrospray source (all Thermo Scientific). A gradient of 6 to 32% acetonitrile–0.1% formic acid over 48 min was used at a flow rate of 0.3 μl/min. The Orbitrap was operated in data-dependent acquisition mode with a survey scan at a resolution of 60,000, and up to the 10 most intense ions were selected for MS/MS. Raw files were processed using Proteome Discoverer (PD), version 1.3 (Thermo Scientific) with Mascot, version 2.4 (Matrix Science, United Kingdom), as the search engine against the appropriate protein database. A decoy database of reversed sequences was used to filter the results at a false detection rate of 1%.

### *K_d_* (dissociation constant) determination for 17-AAG and compound D binding to PfHSP90.

Tryptophan fluorescence analysis was performed using a PerkinElmer LS55 luminescence spectrometer. In order to determine the binding affinity of purified recombinant proteins, 25 μg/ml PfHSP90 or PfHSP90 N-terminal domain (PfHSP90-NTD) was incubated with concentrations of 17-*N*-allylamino-17-demethoxygeldanamycin (17-AAG) or compound D ranging from 0 to 60 μM. The binding buffer used for the reaction contained 50 mM Tris-HCl, pH 7.4, and 1 mM EDTA. Samples were excited at 280 nm, and tryptophan fluorescence measurements were carried out by scanning the emission spectrum in the wavelength range 300 to 400 nm. A maximum wavelength (λ_max_) of 346 nm was selected for all the calculations. The slit widths of excitation and emission were set at 2.5 and 5 nm, respectively. The difference in fluorescence intensity between protein alone and protein with various concentrations of 17-AAG or compound D was calculated and plotted against the different concentrations of the respective ligand used. The resultant hyperbolic curve was analyzed with GraphPad Prism software, using nonlinear regression analysis with single-site-specific binding as described previously (32).

### Docking compound D to PfHSP90.

Docking calculations were performed using the web-based graphical user interface (GUI) of DockingServer by Virtua Drug (Budapest, Hungary) (35). The PfHSP90 N-terminal domain structure was downloaded from the Protein Data Bank (PDB) (PDB accession number 3K60) (36). Chain A of the dimeric PfHSP90 was used to perform docking calculations. Essential hydrogen atoms, Kollman united atom type charges, and solvation parameters were added with the aid of AutoDock tools (37). An affinity map of 20-by-20-by-20-Å grid points, and 0.375-Å spacing was developed using the AutoGrid program (37). Test and control ligand structures were subjected to geometry optimization, and charge calculations were performed using the Merck molecular force field 94 (MMFF94) and Gasteiger methods, respectively (38). Here, Gasteiger partial charges were added to the ligand atoms. Nonpolar hydrogen atoms were merged, and rotatable bonds were defined. AutoDock parameter set and distance-dependent dielectric functions were used in the calculation of the van der Waals and the electrostatic terms, respectively.

The prepared molecular structures were subjected to docking simulation using the Lamarckian genetic algorithm (LGA) and the Solis and Wets local search method (39) which is used by the DockingServer GUI. All rotatable torsions were released during docking. During the search, a translational step of 0.2 Å and torsional steps of 5.0 Å were applied. Docking calculations are the result of 10 autonomous runs. Each run was terminated after a maximum of 250,000 energy evaluations. The population size was set to 150. Here, we used free energy of binding (kcal/mol), number of hydrogen bond interactions, inhibition constant (*K_i_*), and frequency of probable binding sites for ranking our docking results. Frequency shows the percentage of the local searches with similar geometry having a root mean square tolerance (rmstol) of 2 Å. Docked structures with the lowest free energy of binding and *K_i_* values and structures having two or more hydrogen bond interactions between ligand and protein and a frequency of 10% or more were used for prediction of the probable binding configuration for compound D. The analyzed PDB format of the docking file was downloaded and analyzed using Discovery Studio Visualizer, version 4.0.

## RESULTS

### Imidazopyridazines target two distinct stages of asexual parasite development.

**FIG 1:**
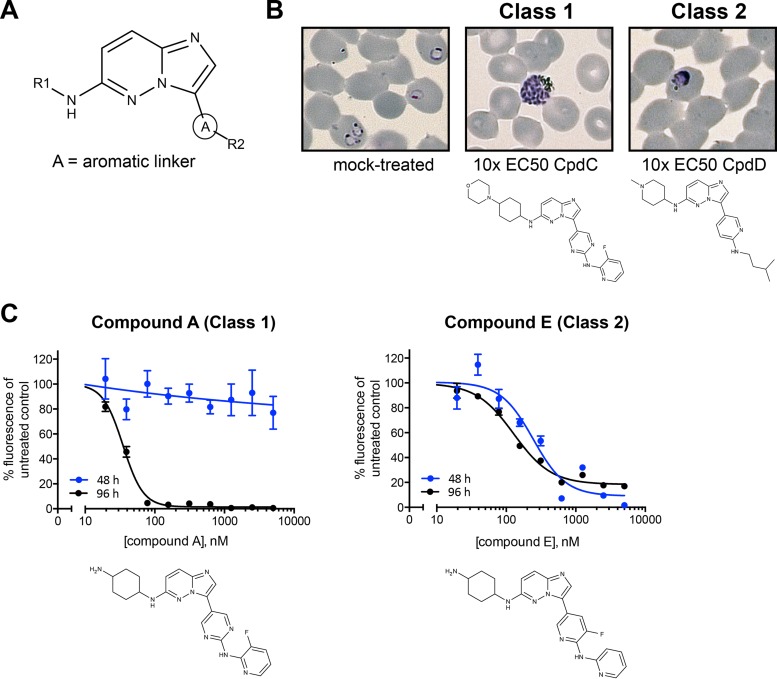
Imidazopyridazine compounds have two modes of action. (A) Imidazopyridazine compounds in this study are characterized by an invariant core, an aromatic linker group (A), and variable R1 and R2 groups. (B) P. falciparum 3D7 parasites approximately 21 h after merozoite invasion of erythrocytes were incubated for 48 h with a concentration equal to 10 times the EC_50_ of each compound (determined by fluorescence-activated cell sorting [23]). In mock-treated cultures (0.05% DMSO), newly invaded erythrocytes with ring stage parasites are seen. In the presence of compound C (CpdC; 0.3 μM), parasites developed until very late schizogony and then arrested prior to merozoite egress, whereas with compound D (CpdD; 4 μM), parasites failed to develop beyond late rings/early trophozoites. (C) EC_50_ determination using a SYBR green I assay. P. falciparum cultures were incubated for either 48 h or 96 h with serial dilutions of inhibitors. For compound A, the EC_50_ with 48 h of incubation was not able to be determined with any accuracy, whereas with 96 h of incubation the EC_50_ was 0.023 μM (standard deviation, 0.007). The EC_50_ for compound E was 0.298 μM (standard deviation, 0.066) or 0.142 μM (standard deviation, 0.024) with 48 h or 96 h of incubation, respectively. The graphs shown are representative examples of triplicate experiments. Error bars show the standard errors of the means for duplicate samples.

Previously, we developed a series of potent CDPK1 inhibitors based on an invariant imidazopyridazine central core, an aromatic linker group (A in Fig. 1A), and variable R1 and R2 groups (Fig. 1A). Some of these compounds inhibit recombinant CDPK1 with low-nanomolar IC_50_s and are extremely effective at killing parasites in culture, with the most potent compound having an EC_50_ of 12 nM (20–23). To determine their modes of action against the parasite, we added the compounds to synchronized parasite populations and identified at which stage they inhibited growth. Examination of Giemsa-stained smears of parasites treated at the ring stage with imidazopyridazines at 10 times their parasiticidal EC_50_s revealed that the compounds had two distinct mechanisms of action during intracellular development. Some (designated class 1 compounds) killed parasites at the mature schizont stage late in the cycle, while others (class 2 compounds) killed parasites at the early trophozoite stage (Fig. 1B). In the former case, while parasites developed into schizonts in the presence of the compounds, they did not rupture the erythrocyte to release merozoites.

As a higher-throughput alternative to microscopic examination, we used a fluorescence-based assay to measure parasite DNA content. Briefly, a trophozoite population collected 21 h after merozoite invasion of erythrocytes was incubated with compound for either 48 or 96 h, representing one or two rounds of parasite replication, respectively. Parasites were then lysed in the presence of SYBR green I, a fluorescent dye that binds preferentially to double-stranded DNA. After 48 h of incubation, parasites treated with compounds that kill following DNA replication are indistinguishable from untreated cells because an arrested schizont will give the same net fluorescence as parasites from a ruptured schizont that have gone on to invade red blood cells. Extending the culture period after drug addition to 96 h enables a true EC_50_ to be measured for all compounds because another round of parasite replication will have occurred in viable parasites. The EC_50_s measured for compounds that act prior to DNA synthesis will be identical regardless of the incubation time, whereas compounds that act after DNA synthesis will appear to be much less potent in the shorter-incubation assay. Use of these two measures enabled compounds to be classified based on the stage at which they killed parasites. Class 1 compounds act after DNA synthesis in late schizogony and prior to merozoite release, whereas class 2 compounds act at the trophozoite stage prior to DNA synthesis. Graphs for exemplar compounds of each class are shown in Fig. 1C.

We analyzed 41 compounds in this manner in order to define the molecular features that determine their modes of action (see Table S1 in the supplemental material). For each compound, the EC_50_s were measured at 48 h (EC_50_48) and 96 h (EC_50_96), and the ratio of the EC_50_s was calculated; an EC_50_48/EC_50_96 ratio greater than 5 was a strong indication that a compound acted after DNA synthesis. When the structure of each compound was examined, a determinant of the stage at which the compound acted was the presence or absence of a pyrimidine at the aromatic linker position (labeled A in Fig. 1A). Of the 16 compounds with a pyrimidine linker, 13 had an EC_50_48/EC_50_96 ratio greater than 5, suggesting that their action was after DNA synthesis (class 1). Of the 25 compounds with nonpyrimidine linkers, 24 had an EC_50_48/EC_50_96 ratio less than 5, suggesting that their action was prior to DNA synthesis (class 2). Full details are provided in Table S1. This classification was again confirmed by microscopic examination of Giemsa-stained blood smears of parasites treated with a subset of 10 compounds (Table 1; see also Table S1 in the supplemental material). Compound 28 was the only class 1 compound shown in Table 1 with an EC_50_48/EC_50_96 ratio less than 5 and was confirmed by microscopy to arrest parasites in late schizogony, indicating that while an EC_50_48/EC_50_96 ratio greater than 5 is suggestive of action after DNA replication, borderline cases need to be confirmed by microscopy. The majority of class 1 compounds (9 of 16) have EC_50_48/EC_50_96 ratios greater than 500.

**TABLE 1:** Compounds divided into two classes based on the aromatic linker

Class and compound^*a*^	Aromatic linker	EC_50_^*b*^ (μM) after treatment for:		EC_50_48/EC_50_96 ratio	Stage of action^*c*^
		48 h	96 h		
Class 1					
28	Pyrimidine	0.168 (0.011)	0.038 (0.012)	4.4	Late schizont
31	Pyrimidine	1.663 (0.650)	0.238 (0.047)	7.0	Late schizont
35	Pyrimidine	>1,000	0.809 (0.451)	>1,000	Late schizont
36 (Cpd A)	Pyrimidine	>1,000	0.023 (0.007)	>1,000	Late schizont
38	Pyrimidine	>1,000	0.050 (0.018)	>1,000	Late schizont
39 (Cpd B)	Pyrimidine	>1,000	0.020 (0.008)	>1,000	Late schizont
41 (Cpd C)	Pyrimidine	>1,000	0.037 (0.005)	>1,000	Late schizont
Class 2					
7 (Cpd D)	Pyridine	0.355 (0.083)	0.311 (0.103)	1.1	Trophozoite
20 (Cpd E)	Fluoropyridine	0.298 (0.066)	0.142 (0.024)	2.1	Trophozoite
22	Pyridine	0.546 (0.080)	0.300 (0.141)	1.8	Trophozoite

a Class 1, pyrimidine linker; class 2, nonpyrimidine linker. Cpd, compound

b EC_50_s of compounds were determined using a SYBR green I assay following treatment of parasite cultures with serial dilutions of the compounds for 48 h or 96 h. Data presented show the mean EC_50_ values from three independent experiments, with the standard deviations shown in parentheses.

c The stage of action was determined by microscopic examination of Giemsa-stained parasite smears after treatment of ring stage parasites with 10 times the 96-h EC_50_ of compound for 48 h.

### Late-acting compounds kill parasites by inhibiting cGMP-dependent kinase (PKG).

We first wished to identify the target of the class 1 inhibitors in the parasite since, although they act very late in schizogony, they may be acting through a target other than CDPK1. For example, the phenotype of parasites treated with purfalcamine, a previously reported CDPK1 inhibitor (15), is very reminiscent of the phenotype of those treated with cGMP-dependent protein kinase (PKG) inhibitors such as “compound 1,” in which merozoites develop within the schizont but are not released because of a defect in egress (40). While it is not impossible that the phenotype of CDPK1 inhibition is indistinguishable from that of PKG inhibition, we wished to definitively identify the primary target(s) of our compounds. CDPK1 and PKG are closely related kinases with regard to ATP binding sites; for example, they share some sequence homology, including a small threonine residue at the gatekeeper position (residue 145 in CDPK1 and residue 618 in PKG). The imidazopyridazine compounds in this study have been shown to interact at the ATP binding site of recombinant CDPK1 *in vitro*, and this binding was blocked by replacing the gatekeeper residue with the larger glutamine, which blocks access to the enzyme's binding pocket, rendering it relatively insensitive to specific inhibitors (23). In a similar chemical genetics approach, the gatekeeper residue of PKG had also been replaced by glutamine (24).

**FIG 2:**
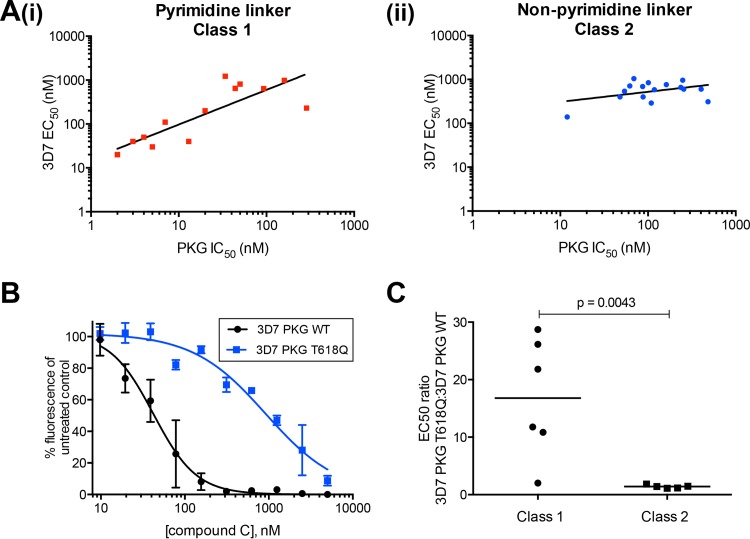
Parasite killing activity of class 1 compounds can be attributed to inhibition of cGMP-dependent protein kinase. (A, panel i) Correlation between P. falciparum EC_50_ and PKG IC_50_ for 13 class 1 compounds. The calculated coefficient of determination (*r*^2^) value of 0.71 from a linear least-squares regression indicates a good correlation between the two data sets. A *P* value of 0.0002 indicates a significant relationship between the two measurements. The correlation between P. falciparum EC_50_ and PKG IC_50_ for 16 class 2 compounds is also shown (panel ii). An *r*^2^ of 0.17 indicates little correlation between the 3D7 EC_50_ and the PKG IC_50_ for class 2 compounds. In addition, a *P* value of 0.114 indicates that there is no significant relationship between the two measures. (B) Parasites expressing a large gatekeeper variant PKG (3D7 PKG T618Q) are insensitive to compound C compared to the sensitivity of WT parasites (3D7 PKG WT). SYBR green assays were used to measure the parasitemia of cultures treated with serial dilutions of compound C for 96 h. The experiment was performed twice; data from a single experiment are shown. The EC_50_s are 0.923 μM (standard deviation, 0.226) for 3D7 PKG T618Q and 0.042 μM (standard deviation, 0.018) for 3D7 PKG WT parasites. Error bars represent the standard errors of the means of duplicate samples. (C) Mann-Whitney test to compare the ratio of EC_50_s for 3D7 PKG T618Q/3D7 WT PKG treated with class 1 and class 2 compounds. Horizontal bars show the median values for each class of compounds (16.8 for class 1 and 1.4 for class 2). The calculated *P* value is 0.0043.

We tested the ability of 30 compounds to inhibit PKG and their sensitivity to a large residue at the gatekeeper position. All of the compounds inhibited recombinant PKG activity, with the most potent compound having an IC_50_ of 1.55 nM (see Table S2 in the supplemental material). We have shown previously that the correlation between the IC_50_ for CDPK1 and the P. falciparum EC_50_ for these compounds was poor (23). When compounds were grouped according to class 1 and 2, there was an excellent correlation between PKG IC_50_ and parasite-killing EC_50_ when the aromatic linker was a pyrimidine (class 1) (Fig. 2A, panel i). When the linker group was not a pyrimidine (class 2), the correlation was poor (Fig. 2A, panel ii). All of the compounds tested against PKG relied on the presence of a small gatekeeper residue for their activity. Replacing threonine 618 with glutamine resulted in an increase in the IC_50_ by a factor of between 20 and over 16,000 (Table 2; see also Table S2 in the supplemental material).

**TABLE 2:** Class 1 compounds inhibit PKG, and their potency against both the enzyme and the parasite is reduced by a larger gatekeeper residue

Class and compound^*a*^	Aromatic linker	Enzyme inhibition (IC_50_ [μM])^*b*^			Selectivity (PKG T618Q/PKG IC_50_ ratio)	Parasite inhibition (EC_50_ [μM])^*c*^		3D7 PKG T618Q/3D7 EC_50_ ratio
		CDPK1	PKG	PKG T618Q		3D7	3D7 PKG T618Q	
Class 1								
28	Pyrimidine	0.012	0.003	31.69	10,563	0.059	0.640	10.8
36 (Cpd A)	Pyrimidine	0.008	0.002	10.96	5,480	0.034 (0.006)	0.901 (0.483)	26.5
38	Pyrimidine	0.008	0.004	43.52	10,880	0.073	0.859	11.8
39 (Cpd B)	Pyrimidine	0.009	0.002	25.54	12,770	0.034	0.982	28.9
41 (Cpd C)	Pyrimidine	0.011	0.013	12.70	977	0.042 (0.018)	0.923 (0.226)	22.0
44	Pyrimidine	0.065	ND	ND		0.236	0.477	2.0
Class 2								
7 (Cpd D)	Pyridine	0.013	0.484	15.51	32	0.427 (0.045)	0.477 (0.180)	1.1
20 (Cpd E)	Fluoropyridine	0.008	0.012	15.33	1,278	0.210	0.386	1.8
22	Pyridine	0.074	>1	37.53	<38	0.215	0.303	1.4
42	Pyridine	0.081	ND	ND		0.836	1.231	1.5
43	Pyridine	0.088	ND	ND		0.080	0.093	1.2
Cpd 1						0.303	1.974	6.5

a Cpd, compound.

b Class 1 pyrimidine-linked compounds in particular display potent inhibition of PKG but not of the T618Q variant. ND, not determined.

c Parasite inhibition data are calculated from a single experiment, other than duplicate assays performed on compounds A, C, and D. For these compounds, the mean EC_50_ values are shown, with the standard deviations from the means in parentheses.

To establish whether PKG was the primary target of the inhibitors in the parasite, we used a parasite line expressing a variant PKG T618Q enzyme (24) and measured the EC_50_ of six class 1 and five class 2 compounds against 3D7 wild-type (WT) and PKG T618Q parasites, using the SYBR green I assay described previously, with a 96-h incubation time. We also included compound 1, a trisubstituted pyrrole previously shown to be an apicomplexan PKG inhibitor that is also sensitive to the size of the gatekeeper residue (24). All but one of the class 1 compounds had decreased parasiticidal activity against the PKG T618Q variant parasite, with compound B (a class 1 compound) showing the greatest effect, a 29-fold decrease in potency (Table 2 and Fig. 2B). The exception is compound 44, a class 1 compound with an isobutyl aliphatic group at position R2, whereas the other compounds have fluorophenyl R2 groups. It is possible that the smaller aliphatic R2 group enables this compound to bind to the modified enzyme, despite the large gatekeeper residue. Examination of Giemsa-stained smears of 3D7 parasites treated at the ring stage with compound 44 at its EC_90_ (0.65 μM) showed that the parasites were arrested at the schizont stage, suggesting that it behaves like other class 1 compounds in terms of its mode of parasite killing. Compound 1 showed a 7-fold decrease in potency against the PKG T618Q parasite, in line with previously published data (24, 40). In contrast, none of the class 2 compounds (nonpyrimidine linker group) showed a significant increase in the EC_50_ against the PKG T618Q parasite, (Table 2; see also Table S2 in the supplemental material). To determine the statistical significance of the difference between the two classes of compounds, a Mann-Whitney test was performed. The median ratio of the EC_50_ for 3D7 PKG T618Q/EC_50_ for 3D7 of class 1 compounds was 16.8, and for class 2 compounds the median EC_50_ ratio was 1.4. The *P* value was calculated to be 0.0043, indicating that the difference between these two classes of compounds is significant (Fig. 2C). Taken together, these data strongly suggest that the class 1 compounds exert their parasiticidal effect primarily through inhibition of PKG activity, whereas the class 2 compounds do not.

### Early-acting compounds may kill parasites by inhibiting HSP90.

**FIG 3:**
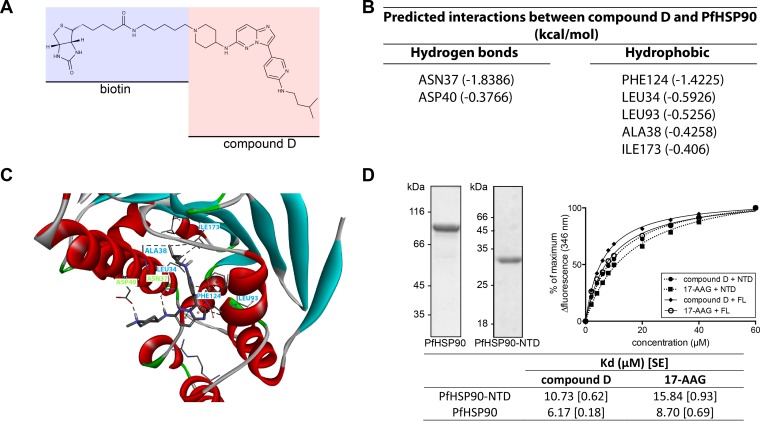
Affinity purification of cellular targets of compound D. (A) Biotin linked to the R1 group of compound D. This compound was bound to streptavidin-agarose and used to affinity purify proteins from a trophozoite cell lysate. The only significant hit identified by LC-MS/MS was HSP90 (see Table S3 in the supplemental material). (B) Predicted interactions between compound D and PfHSP90. (C) Modeling of the most likely binding orientation of compound D to the ATP binding site of HSP90 was carried out using DockingServer. Residues predicted to form hydrogen bonds with compound D are labeled in green, while those predicted to form hydrophobic interactions are labeled in blue. (D) Recombinant PfHSP90 binds to compound D. Purified recombinant PfHSP90 and PfHSP90-NTD used in subsequent experiments are shown in the Coomassie-stained gels to the left of the figure. Changes in the tryptophan fluorescence (346 nm) of PfHSP90 were monitored in the presence of increasing amounts of compound D or 17-AAG. *K_d_* values were calculated for both full-length (FL) PfHSP90 and PfHSP90-NTD. Values in brackets are the standard errors (SE) of the means of triplicate measurements.

The early stage of the asexual cycle targeted by class 2 compounds precludes the target from being CDPK1 as it is not expressed in such early parasites (41–43). To identify their target, we selected an exemplar class 2 compound, compound D, resynthesized it with biotin attached to the R1 group (Fig. 3A), and made an affinity resin using streptavidin agarose. We purified interacting proteins from a lysate prepared from infected erythrocytes approximately 24 h after invasion by merozoites and identified interacting proteins by LC-MS/MS. For proteins to be defined as hits they had to be present in replicate LC-MS/MS analyses with compound D resin and absent from duplicate control resin assays, one with unloaded streptavidin and another where the biotin was attached to the R2 group.

Most of the proteins detected were very weak hits, with fewer than five unique peptides identified. The only exception was HSP90 (PF3D7_0708400), which had 24 unique peptide hits (see Table S3 in the supplemental material). HSP90 is a protein chaperone with an ATP binding site in its N-terminal domain. Using *in silico* methods, we judged that the free energy of binding of compound D to the ATP binding pocket of PfHSP90 was favorable; Fig. 3B shows the residues of PfHSP90 predicted to interact with compound D, and Fig. 3C shows the most likely binding orientation of the compound. From the docking simulation it can be predicted that compound D will form both polar and hydrophobic interactions with the amino acid residues in the binding pocket of PfHSP90. Our analysis suggests that the nitrogen atom of the piperidine group may attain a quaternary state to form a hydrogen bond interaction with the carboxyl group of Asp40. In the given orientation, the amino linker between the imidazopyridazine and piperidine groups might also form a hydrogen bond interaction with Asn37. Further, the pyridine (aromatic linker) in compound D could form a mixed pi-alkyl hydrophobic interaction with Leu93. We found that the amino alkyl branched chain attached to the aromatic linker may also interact with Leu34, Ala38, and Ile173 to promote the formation of a stable protein-ligand complex. In addition, Phe124 may interact with the imidazopyridazine nucleus of compound D by both pi and alkyl hydrophobic interactions. The Gibbs binding free energy for the selected orientation of compound D in the binding pocket of PfHSP90 was found to be −7.42 kcal/mol, with a root mean square tolerance (rmstol) of 2 Å. It is well established that the *K_d_* value of a compound depends on the Gibbs binding free energy (44). The *K_i_* value for the compound was predicted to be 3.64 μM. The interaction of compound D with recombinant PfHSP90 and the N-terminal domain of HSP90 (PfHSP90-NTD) *in vitro* was confirmed by measuring changes in intrinsic tryptophan fluorescence values upon binding (32). The *K_d_* values obtained for compound D binding to full-length PfHSP90 and PfHSP90-NTD were 6.17 and 10.73 μM, respectively. In both cases the data indicated a slightly stronger interaction than that of 17-AAG, an established HSP90 inhibitor (45) (Fig. 3D).

### Inhibition of CDPK1 has no effect on asexual parasite development.

**FIG 4:**
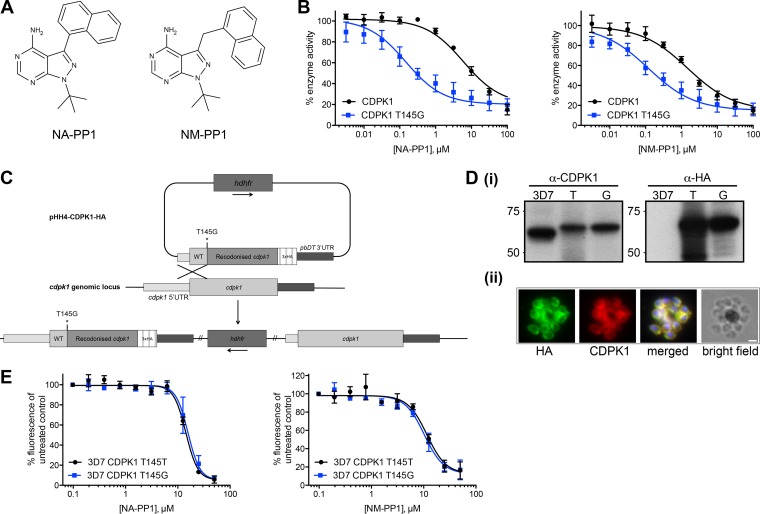
Inhibition of PfCDPK1 has no effect on asexual parasite growth. (A) Chemical structures of the bumped kinase inhibitors (BKIs) NA-PP1 and NM-PP1. (B) Recombinant CDPK1 with a glycine at the gatekeeper residue (amino acid 145) shows increased sensitivity to both BKIs. A ParM ADP biosensor assay to measure ATPase activity of CDPK1 enzymes was carried out in the presence of serial dilutions of BKIs. The IC_50_ of CDPK1 T145G for NA-PP1 decreased 41-fold compared to that of WT CDPK1, with IC_50_s of 0.15 μM (95% confidence interval, 0.08 to 0.26 μM) and 6.01 μM (95% confidence interval, 3.4 to 10.5 μM), respectively. The IC_50_ of NM-PP1 was 0.12 μM (95% confidence interval, 0.06 to 0.23 μM) for CDPK1 T145G compared to 1.5 μM (95% confidence interval, 0.77 to 2.90 μM) for WT CDPK1, a 13-fold decrease. Graphs show mean values from five independent experiments, with error bars indicating the standard errors of the means. (C) Scheme for generating a P. falciparum 3D7 CDPK1 T145G-HA parasite line by single-crossover homologous recombination at the *cdpk1* genomic locus. Crossover upstream of the T145 codon is forced by recodonizing sequences downstream of and including the T145 codon. Integration at the locus results in a chimeric *cdpk1* gene with a modified gatekeeper residue. An identical method was used in which the gatekeeper residue was left as a threonine to generate the parasite line 3D7 CDPK1 T145T-HA. UTR, untranslated region. (D, panel i) Whole-cell lysates from P. falciparum 3D7, 3D7 CDPK1 T145T-HA (T), and 3D7 CDPK1 T145G-HA (G) were probed with antibodies against CDPK1 and against the HA epitope tag. Immunofluorescence using anti-HA and anti-CDPK1 antibodies confirms that CDPK1 T145G-HA localizes correctly to the plasma membrane of parasites (panel ii). Scale bar, 1 μm. (E) SYBR green assays to measure the EC_50_s of NA-PP1 and NM-PP1 for P. falciparum 3D7 CDPK1 T145T-HA and 3D7 CDPK1 T145G-HA lines. The EC_50_ for NA-PP1 for 3D7 CDPK1 T145T-HA was 14.26 μM (95% confidence interval, 12.3 to 16.5 μM), and that for 3D7 CDPK1 T145G-HA was 15.65 μM (95% confidence interval, 13.4 to 18.3 μM). The EC_50_s for NM-PP1 were 11.55 μM (95% confidence interval, 9.1 to 14.6 μM) and 10.28 μM (95% confidence interval, 8.1 to 13.0 μM) for 3D7 CDPK1 T145T-HA and 3D7 CDPK1 T145G-HA, respectively. The experiments were performed three times, with at least six replicate samples per experiment. Graphs show mean values from three independent experiments, with error bars indicating the standard errors of the means.

To unequivocally determine the effect of inhibiting CDPK1 on asexual blood stage parasite development, we adopted a chemical genetics approach. Modification of the gatekeeper residue of a kinase can alter its sensitivity to a group of large inhibitors known as bumped kinase inhibitors (BKIs). Kinases with large gatekeeper residues tend to be insensitive to these inhibitors, whereas those with small gatekeeper residues are more sensitive (46, 47). The CDPK1 T145G enzyme, which has the smallest amino acid—glycine—at this position, has kinase activity in line with that of the wild-type enzyme and is sensitive to inhibition by the imidazopyridazine compounds (23). We tested two chemically related BKIs, NA-PP1 and NM-PP1, which are identical except for the extended methylene linker of NM-PP1, which causes the orientation of the napthyl group to change relative to the pyrazolopyrimidine core (Fig. 4A). Both of these compounds are reversible, cell-permeable inhibitors of small gatekeeper residue kinases and have been used in both mammalian (48, 49) and Saccharomyces cerevisiae cells (47, 50). CDPK1 T145G was 41 times more sensitive than the wild-type enzyme to inhibition by NA-PP1, with IC_50_s of 0.15 and 6.01 μM, respectively, and 13 times more sensitive to NM-PP1 than the wild-type enzyme, with IC_50_s of 0.12 and 1.50 μM, respectively (Fig. 4B). We used statistical analysis within GraphPad Prism to compare the individual curve fits for each data set with a curve fit to all the data sets, with a null hypothesis that the IC_50_s are the same for both data sets. An extra-sum-of-squares *F* test was used to compare the goodness of fit of the two alternative models. A *P* value of <0.0001 indicated that one curve did not fit to both data sets as well as individual curves and that the IC_50_s are therefore significantly different for both inhibitors with the two variant enzymes.

Having established this differential sensitivity of a gatekeeper variant CDPK1, we generated by homologous recombination at the endogenous gene locus a parasite line that expressed CDPK1 T145G. The transgenic parasite was engineered to also express a triple HA tag at the extreme C terminus of the enzyme. A control parasite line was made using an identical targeting approach in which a triple HA tag was added to the protein, but no amino acid substitution was introduced at the gatekeeper residue (Fig. 4C). Using an anti-HA antibody on Western blots of schizont lysates from the parasite lines, the modified proteins were the expected size, and there was no evidence of unmodified protein in either line (Fig. 4D, panel i). The T145G variant protein was expressed in late-stage schizonts and targeted to the periphery of merozoites in the same way as the wild-type enzyme (Fig. 4D, panel ii). The two parasite lines were incubated with NA-PP1 and NM-PP1, and the EC_50_s for each compound were calculated (Fig. 4E). The EC_50_s for NA-PP1 were 14.26 μM for 3D7 CDPK1 T145T and 15.65 μM for 3D7 CDPK1 T145G. For NM-PP1 the EC_50_s were 11.55 μM and 10.28 μM for 3D7 CDPK1 T145T and 3D7 CDPK1 T145T parasite lines, respectively. An extra-sum-of-squares *F* test to compare the fit of one curve to both data sets with that of individual curves for each data set, with a null hypothesis that the same curve could fit both data sets, gave a *P* value of 0.70 for NA-PP1 treatment of the two parasite lines and of 0.79 for NM-PP1, indicating that the same curve could be used to fit the data for both parasite lines with the inhibitors and that there is no significant difference between the sensitivities of the parasite lines to either of the BKIs. These data suggest that inhibition of CDPK1 has no effect on asexual parasite viability.

## DISCUSSION

The need for novel drugs against malaria has resulted in the emergence of protein kinases as potential new targets (7). While little is known about their detailed role in the biology of the parasite, evidence from reverse genetics approaches regarding the essentiality of a kinase has guided target selection (12). One such kinase is PfCDPK1, which had been thought to be essential in asexual blood stages due to an inability to disrupt the gene in P. falciparum and P. berghei (15, 16).

We have characterized a series of imidazopyridazines that were developed as CDPK1 inhibitors (20–23). We show here that the parasiticidal activity of the compounds falls into two distinct classes based on the timing of action during the asexual cycle of P. falciparum. Furthermore, we established that the stage in the asexual cycle at which the compounds act is governed by the nature of the aromatic linker group. Class 1 compounds with a pyrimidine ring at this position killed parasites at late schizogony by inhibition of PKG. Class 2 compounds with a nonpyrimidine moiety at this position caused parasite death at the trophozoite stage, probably by inhibiting the activity of HSP90. It is remarkable that the mode of action of the inhibitors is changed by relatively small changes in the aromatic linker group.

We have demonstrated that the primary target of class 1 imidazopyridazines is PKG, and the consequence of this inhibition is a failure of merozoites to egress from schizonts. Although we had shown previously that CDPK1 is potently inhibited by these compounds (23), it appears that inhibition of CDPK1 has no effect on parasite development: when an effect on PKG is discounted by using a parasite expressing an inhibitor-insensitive PKG, the parasite's sensitivity to the compounds is very substantially reduced. If inhibition of CDPK1 contributed to the observed effect on merozoite egress, then we would have expected the PKG T618Q parasite line to retain sensitivity to these compounds, which it did not.

The identification of the protein chaperone HSP90 by affinity purification with compound D was unexpected as the compounds had been developed as kinase inhibitors. However, HSP90 has essential ATPase activity, and it is likely that the class 2 imidazopyridazine compounds bind to the ATP-binding site of both HSP90 and CDPK1 even though these binding sites are quite dissimilar. It is possible that the inhibitory phenotype seen in trophozoites in the presence of class 2 compounds may be caused by the inhibition of kinases or ATPases, in addition to HSP90, that were not identified in the affinity purification using compound D. However, it remains that HSP90 is a most promising candidate based on the experiments we have presented here. The binding of compound D to HSP90 was confirmed experimentally. The use of HSP90 inhibitors to block malaria parasite development is not a new concept. Geldanamycin is a benzoquinone ansamycin known to be an ATP-competitive inhibitor of human HSP90 (51). Both geldanamycin and its derivative 17-AAG have been shown to block parasite development at the trophozoite stage and to inhibit the ATPase activity of P. falciparum HSP90 (32, 52). Another HSP90 inhibitor is the purine analogue, PU-H71, which has antiparasite activity *in vitro* and *in vivo* and displays a significant synergistic effect with chloroquine (53). A series of 7-azaindole compounds show exquisite binding selectivity for the parasite HSP90 over the human isoforms and have been shown to inhibit P. falciparum growth in culture (54). The class 2 imidazopyridazines represent a new starting point to generate Plasmodium-specific HSP90 inhibitors.

Using a chemical genetics approach, we produced a P. falciparum line that expresses a CDPK1 gatekeeper variant that displays increased sensitivity to bumped kinase inhibitors. There was no difference in the sensitivities to either BKI of this parasite line and one expressing an unchanged CDPK1. This supports the notion that inhibiting CDPK1 may not have an effect on parasite viability and that the imidazopyridazine compounds kill parasites by inhibition of other enzymes, despite their extremely potent inhibition of CDPK1 activity *in vitro* (and presumably *in vivo*). Both of the BKIs used in this study have been demonstrated to be cell permeable in several other systems (47–50), so it is reasonable to presume that P. falciparum is also accessible to inhibition with these compounds. Similar chemical genetics approaches have been adopted to study the orthologue of CDPK1 in Toxoplasma gondii, TgCDPK3. Lourido and colleagues identified subtle differences in gliding motility when parasites expressing TgCDPK3 with a glycine gatekeeper residue were treated with BKIs, and the ability to respond to ionophore-induced increases in intracellular calcium was impaired (55). There are differences in parasite biology and likely mechanisms of egress between T. gondii and P. falciparum, and therefore direct extrapolation of results from either system to the other may be unwise (56).

Our view that CDPK1 may not be essential in P. falciparum asexual blood stages is supported by recent studies in P. berghei. Sebastian and colleagues showed that when a promoter-swap approach to express P. berghei CDPK1 (PbCDPK1) at almost undetectable levels in asexual blood stages was used, there was no effect on parasite development during these stages (9). More recently, while our investigations were ongoing, a study that knocked out the *Pbcdpk1* gene demonstrated conclusively that PbCDPK1 is entirely dispensable in blood stages, with no effect on any stage of asexual parasite development (17). There is no reason to think that CDPK1 plays different roles in P. falciparum and P. berghei; indeed *Pfcdpk1* can substitute for *Pbcdpk1* with no obvious effect on P. berghei at any stage of development (R. Tewari, personal communication).

Purfalcamine, a 2,6,9-trisubstituted purine, has been identified as an inhibitor of PfCDPK1, and in its presence P. falciparum parasites accumulate in late schizogony, with a block of merozoite egress (15). While there is no doubt that purfalcamine potently inhibits recombinant CDPK1, it is possible that it exerts its parasiticidal effect by inhibiting enzymes other than CDPK1, such as PKG, or perhaps other CDPKs, such as CDPK4 or CDPK5, which is known to be involved in merozoite egress (57). It will be important to establish whether the parasiticidal effect of purfalcamine is sensitive to the size of the PKG gatekeeper residue.

Target validation for drug discovery can be a complex process, and extrapolating from genetic knockout studies to the consequences of inhibition by small molecules can be problematic (58). Where the phenotype of a genetic knockout is in doubt, target-based drug discovery can lead to the erroneous attribution of off-target activities of compounds to the protein of interest. The evidence we have presented casts considerable doubt on the suitability of CDPK1 as a drug target for blood stage Plasmodium infections. While CDPK1 may represent a target for transmission-blocking treatment, owing to its proven role in the sexual stages of the parasite (9), it seems that inhibition of the enzyme during the asexual cycle causes no measurable reduction in fitness of the parasite, and as such CDPK1 may not be worth pursuing as a drug target for the asexual stages of Plasmodium.

## Supplementary Material

Supplemental material
