# Supplementary material for: Imidazopyridazine Inhibitors of Plasmodium falciparum Calcium-Dependent Protein Kinase 1 Also Target Cyclic GMP-Dependent Protein Kinase and Heat Shock Protein 90 To Kill the Parasite at Different Stages of Intracellular Development
Source: Antimicrob Agents Chemother. 2016 Feb 26;60(3):1464–75. doi: 10.1128/AAC.01748-15 (PMC4775997; doi:10.1128/AAC.01748-15)
Supplement: Supplemental material [file supp_60_3_1464__index.html]

Supplemental material 

# Imidazopyridazine inhibitors of *Plasmodium falciparum* calcium dependent protein kinase 1 also target cGMP-dependent protein kinase and heat shock protein 90 to kill the parasite at different stages of intracellular development.

## Supplemental material

- Supplemental file 1 -

  Supplemental information and Tables S1 to S3.

  PDF, 299K
