# Supplementary material for: Imidazopyridazine Inhibitors of Plasmodium falciparum Calcium-Dependent Protein Kinase 1 Also Target Cyclic GMP-Dependent Protein Kinase and Heat Shock Protein 90 To Kill the Parasite at Different Stages of Intracellular Development
Source: Antimicrob Agents Chemother. 2016 Feb 26;60(3):1464–75. doi: 10.1128/AAC.01748-15 (PMC4775997; doi:10.1128/AAC.01748-15)
Supplement: Supplemental material [file AAC.01748-15_zac003164909so1.pdf]

Supplementary Information 1: Synthesis of biotinylated compound D

5-[(3aS,4S,6aR)-2-Oxo-1,3,3a,4,6,6a-hexahydrothieno[3,4-d]imidazol-4-yl]-N-[5-[4-[[3-[6-(isopentylamino)-3-pyridyl]imidazo[1,2-b]pyridazin-6-yl]amino]-1-piperidyl]pentyl]pentanamide

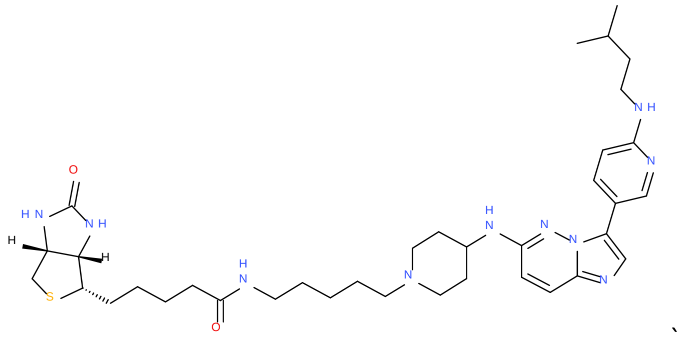

***tert*-Butyl 4-[(3-bromoimidazo[1,2-b]pyridazin-6-yl)amino]piperidine-1-carboxylate**

A solution of 3-bromo-6-chloroimidazo[1,2-b]pyridazine (3.00 g, 12.9 mmol 1.0 eq) in NMP (5 mL) was treated with *tert*-butyl 4-aminopiperidine-1-carboxylate (5.10 g, 25.8 mmol, 2.0 eq), DIPEA (5.60 mL, 32.3 mmol, 2.5 eq) and heated at 140°C for 8 hours. The reaction mixture was diluted with EtOAc (100 mL) and washed with de-ionised water (4 x 100 mL) and brine (100mL). The separated organic was dried ($MgSO_4$), concentrated *in vacuo* and column chromatography (50-100% EtOAc in pet. ether) gave an off-white solid (2.07 g, 40%); ^{1}H NMR (400 MHz, DMSO-d_6) δ = 7.71 (d, *J*=9.6 Hz, 1 H), 7.48 (s, 1 H), 7.10 (d, *J*=7.3 Hz, 1H), 6.68 (d, *J*=10.1 Hz, 1H), 3.87-3.82 (m, 3H), 3.02-2.93 (m, 2H), 2.02-1.98 (m, 2H), 1.41 (s, 9H), 1.40-1.31 (m, 2H); m/z (ES+APCI)$^+$: 396/398 $[M+H]^+$.

3-[6-(Isopentylamino)-3-pyridyl]-N-(4-piperidyl)imidazo[1,2-b]pyridazin-6-amine

A mixture of *tert*-butyl 4-[(3-bromoimidazo[1,2-b]pyridazin-6-yl)amino]piperidine-1-carboxylate (1.00 g, 2.52 mmol, 1.0 eq), 2-isopentylamino-5-pyridineboronic acid pinacol ester (876 mg, 3.02 mmol, 1.2 eq), Pd(dppf)Cl_2 (238 mg, 0.29 mmol, 0.1 eq), Cs_2CO_3 (3.28 g, 10.08 mmol, 4.0 eq), water (1 mL) and THF (10 mL) was heated at 90°C for 4 hours. The mixture was allowed to cool, then concentrated to dryness and purified by chromatography on silica gel (50-100% EtOAc in pet. ether) to give 223 mg of the protected intermediate which was treated with 4M HCl in Dioxane and purification by prep-HPLC gave the product as a white solid (140 mg, 15%); ^{1}H NMR (400MHz, DMSO-d_6) δ = 8.70 (d, *J*=2.3 Hz, 1H), 8.07 (dd, *J*=2.5, 8.9 Hz, 1H), 7.69 (d, *J*=9.6 Hz, 1H), 7.67 (s, 1H), 6.93 (d, *J*=6.9 Hz, 1H), 6.67-6.63 (m, 1H), 6.61 (d, *J*=9.6 Hz, 1H), 6.52 (d, *J*=8.2 Hz, 1H), 3.76-3.58 (m, 1H), 3.34-3.22 (m, 2H),

3.07-2.96 (m, 2H), 2.65-2.55 (m, 2H), 2.07-1.95 (m, 2H), 1.76-1.57 (m, 1H), 1.51-1.40 (m, 2H), 1.40-1.29 (m, 2H), 0.91 (d, *J*=6.9 Hz, 6H); m/z $(ES+APCI)^+$: 380 $[M+H]^+$.

Benzyl N-(5-bromopentyl)carbamate

To a solution of benzyl N-(5-hydroxypentyl)carbamate (1.00 g, 4.21 mmol, 1.0 eq) in DCM was added tetrabromomethane (1.53 g, 4.63 mmol, 1.1 eq) and triphenylphosphine (2.20 g, 8.42 mmol, 2.0 eq) and the reaction mixture was stirred overnight at RT. The solution was concentrated *in vacuo* and purification by column chromatography (10-50% EtOAc in pet. ether) gave the product as a white solid (1.10 g, 87%); ^{1}H NMR (400MHz, $CDCl_3$) δ = 7.42-7.27 (m, 5H), 5.08 (s, 2H), 4.77 (br. s, 1H), 3.39 (t, *J*=6.6 Hz, 2H), 3.20 (q, *J*=6.4 Hz, 2H), 1.86 (quin, *J*=7.0 Hz, 2H), 1.59-1.39 (m, 4H).

N-[1-(5-Aminopentyl)-4-piperidyl]-3-[6-(isopentylamino)-3-pyridyl]imidazo[1,2-b]pyridazin-6-amine

To a solution of 3-[6-(isopentylamino)-3-pyridyl]-N-(4-piperidyl)imidazo[1,2-b]pyridazin-6-amine (140 mg, 0.368 mmol, 1.0 eq) in EtOH (2 mL) was added Et_3N (102 µL, 0.736 mmol, 2.0 eq) and benzyl *N*-(5-bromopentyl)carbamate (110 mg, 0.368 mmol, 1.0 eq) and stirred overnight at RT. The reaction mixture was concentrated *in vacuo* and purification by column chromatography (5-25% MeOH in DCM) to give the protected intermediate which was hydrogenated on an H-Cube (10% Pd/C, Full H2, 50°C) to give the product as an off-white solid (44 mg, 26%); ^{1}H NMR (400MHz, DMSO-d_6) δ = 8.73 (s, 1H), 8.72 (s, 1H), 8.05 (d, *J*=8.2 Hz, 1H), 7.71-7.67 (m, 1H), 7.67 (s, 1H), 6.92 (d, *J*=6.2 Hz, 1H), 6.66 (t, *J*=5.7 Hz, 1H), 6.61 (d, *J*=9.6 Hz, 1H), 6.52 (d, *J*=9.0 Hz, 1H), 4.10 (m, 2H), 3.58 (m, 1H), 3.37-3.26 (m, 2H), 2.85 (d, *J*=11.4 Hz, 2H), 2.75-2.70 (m, 2H), 2.28-2.25 (m, 2H), 2.08-1.94 (m, 4H), 1.71-1.62 (m, 1H), 1.54-1.39 (m, 8H), 1.35-1.23 (m, 2H), 0.92 (d, *J*=6.9 Hz, 6H).

5-[(3aS,4S,6aR)-2-Oxo-1,3,3a,4,6,6a-hexahydrothieno[3,4-d]imidazol-4-yl]-N-[5-[4-[[3-[6-(isopentylamino)-3-pyridyl]imidazo[1,2-b]pyridazin-6-yl]amino]-1-piperidyl]pentyl]pentanamide

To a solution of biotin (21 mg, 0.086 mmol, 1.0 eq) in DMF (1 mL) and Et_3N (60 µL, 0.43 mmol, 5.0 eq) was added TBTU (30 mg, 0.095 mmol, 1.1 eq) and the mixture stirred at RT for 10 minutes before adding *N*-[1-(5-aminopentyl)-4-piperidyl]-3-[6-(isopentylamino)-3-pyridyl]imidazo[1,2-b]pyridazin-6-amine (44 mg, 0.095 mmol, 1.1 eq). The reaction mixture was stirred for 2 hours at RT, concentrated *in vacuo* and purification by prep-HPLC gave the product as a yellow solid (26 mg, 40%); ^{1}H NMR (400MHz, DMSO-d_6) δ = 8.71 (s, 1H), 8.06 (d, *J*=8.2 Hz, 1H), 7.77-7.66 (m, 3H), 6.92 (d, *J*=6.0 Hz,

1H), 6.66 (t, *J*=5.5 Hz, 1H), 6.61 (d, *J*=10.1 Hz, 1H), 6.52 (d, *J*=8.9 Hz, 1H), 6.43 (s, 1H), 6.36 (s, 1H), 4.31-4.27 (m, 1H), 4.14-4.09 (m, 1H), 3.55 (m, 1H), 3.33-3.30 (m, 2H), 3.12-2.99 (m, 3H), 2.81 (m, 3H), 2.59-2.52 (m, 1H), 2.26 (br. s, 2H), 2.11-1.95 (m, 6H), 1.71-1.53 (m, 2H), 1.52-1.36 (m, 11H), 1.34-1.23 (m, 4H), 0.92 (d, *J*=6.4 Hz, 6H); m/z $(ES+APCI)^+$: 691 $[M+H]^+$.

5-[(3aS,4S,6aR)-2-Oxo-1,3,3a,4,6,6a-hexahydrothieno[3,4-d]imidazol-4-yl]-N-[4-[[5-[6-[(1-methyl-4-piperidyl)amino]imidazo[1,2-b]pyridazin-3-yl]-2-pyridyl]amino]butyl]pentanamide

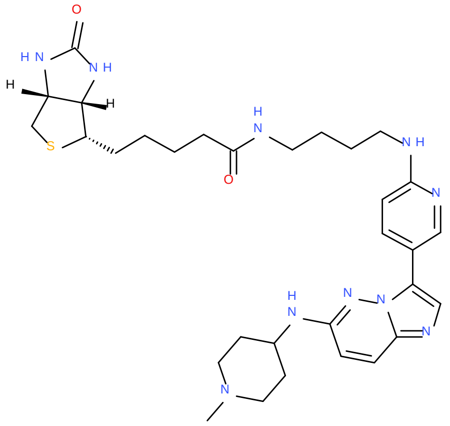

3-(6-Chloro-3-pyridyl)-N-(1-methyl-4-piperidyl)imidazo[1,2-b]pyridazin-6-amine

A mixture of 3-bromo-N-(1-methyl-4-piperidyl)imidazo[1,2-b]pyridazin-6-amine (1.00 g, 3.22 mmol, 1.0 eq), (6-chloro-3-pyridyl)boronic acid (608 mg, 3.86 mmol, 1.2 eq), Pd(dppf)Cl_2 (262 mg, 0.32 mmol, 0.1 eq), Cs_2CO_3 (4.2 g, 12.88 mmol, 4.0 eq), water (1 mL) and THF (10 mL) was combined and heated at 80°C for 4 hours. The mixture was allowed to cool, then concentrated to dryness and purified by column chromatography (50-100% EtOAc in pet. ether) to give the crude product as a yellow solid (853 mg, 77%) which was used without further purification; ^{1}H NMR (400MHz, DMSO-d_6) δ = 9.21 (d, *J*=3.2 Hz, 1H), 8.55 (dd, *J*=2.5, 8.5 Hz, 1H), 8.02 (s, 1H), 7.76 (d, *J*=9.6 Hz, 1H), 7.58 (d, *J*=9.2 Hz, 1H), 7.11 (d, *J*=6.3 Hz, 1H), 6.74-6.63 (m, 1H), 3.65-3.48 (m, 1H), 2.85-2.68 (m, 2H), 2.21 (s, 3H), 2.19-2.07 (m, 2H), 2.05-1.94 (m, 2H), 1.56-1.39 (m, 2H).

5-[(3aS,4S,6aR)-2-Oxo-1,3,3a,4,6,6a-hexahydrothieno[3,4-d]imidazol-4-yl]-N-[4-[[5-[6-[(1-methyl-4-piperidyl)amino]imidazo[1,2-b]pyridazin-3-yl]-2-pyridyl]amino]butyl]pentanamide

To a solution of $Pd(OAc)_2$ (4 mg, 0.014 mmol, 0.5 eq) in DME (0.5 mL) was added CyPF^{t}Bu (8 mg, 0.014 mmol, 0.5 eq). The solution was allowed to stand for 2 minutes then added to a solution of 3-(6-chloro-3-pyridyl)-*N*-(1-methyl-4-piperidyl)imidazo[1,2-b]pyridazin-6-amine (100 mg, 0.291 mmol, 1.0

eq) and NaO^tBu (39 mg, 0.408 mmol, 1.4 eq) in DME (1.5 mL). The reaction mixture was stirred for 5 minutes before adding *tert*-butyl *N*-(4-aminobutyl)carbamate (78 µL, 0.408 mmol, 1.4 eq) and stirring for a further 2 hours at 90°C. The reaction mixture was concentrated *in vacuo* and purification by column chromatography (0-10% MeOH in DCM with 1% NH_4OH) gave *tert*-butyl *N*-[4-[[5-[6-[(1-methyl-4-piperidyl)amino]imidazo[1,2-b]pyridazin-3-yl]-2-pyridyl]amino]butyl]carbamate as a yellow solid (122 mg, 0.247 mmol, 1.0 eq) which was treated with 4 M HCl in dioxane (1 mL). The reaction mixture was stirred at RT for 2 hours before concentrating *in vacuo*. The resulting solid was dissolved in DMF (1 mL) and treated with Et_3N (178 µL, 1.235 mmol, 5.0 eq) then added to a pre-mixed solution of biotin (72 mg, 0.296 mmol, 1.2 eq) and TBTU (95 mg, 0.296 mmol, 1.2 eq) in DMF (1 mL). The reaction mixture was stirred overnight at RT, concentrated *in vacuo* and purification by prep HPLC gave the product as an off-white solid (8 mg, 5%); ^1H NMR (400MHz, DMSO-d_6) δ = 8.68 (d, *J*=2.3 Hz, 1H), 8.09 (dd, *J*=2.3, 8.8 Hz , 1H), 7.77 (t, *J*=5.6 Hz, 1H), 7.70 – 7.67 (m, 2H), 6.91 (d, *J*=6.1 Hz, 1H), 6.70 (t, *J*=5.4 Hz, 1H), 6.61 (d, *J*=9.8 Hz, 1H), 6.53 (d, *J*=8.9 Hz, 1H), 6.42 (s, 1H), 6.35 (s, 1H), 4.29-4.25 (m, 1H), 4.16-4.04 (m, 1H), 3.57 – 3.53 (m, 1H), 3.28-3.25 (m, 2H), 3.10-2.98 (m, 3H), 2.82-2.72 (m, 3H), 2.59-2.52 (m, 1H), 2.18 (s, 3H), 2.08-1.98 (m, 6H), 1.63-1.40 (m, 10H), 1.37-1.23 (m, 2H); m/z $(ES+APCI)^+$: 621 $[M+H]^+$.

Table S1. EC_{50} values of compounds determined using the SYBR Green I assay following treatment of parasite cultures with serial dilutions of the compounds for 48 h or 96 h, and stage of action determined by microscopy for a subset of compounds. Values are from a single experiment with duplicate samples for each compound, unless indicated by the presence of the standard deviation of the mean in parentheses after the EC_{50} value. Where this is present, the EC_{50} given is the mean of triplicate independent experiments.

Compound	Structure	Aromatic linker	EC_{50} (µM)		ratio $EC_{50}48:EC_{50}96$	[a]Stage of action (before or after DNA synthesis)
			48 hours	96 hours		
1	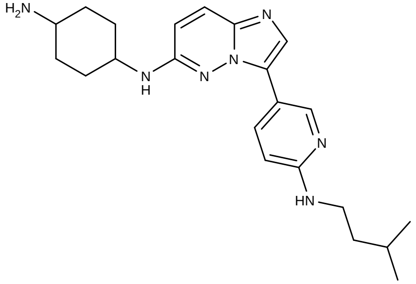	pyridine	0.29	0.29	1.0	
2	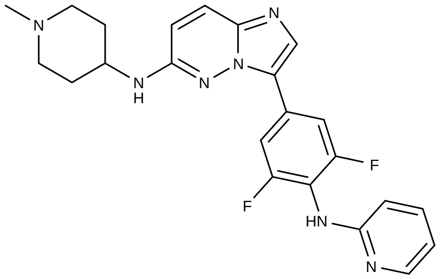	difluorophenyl	1.28	1.04	1.2	

3	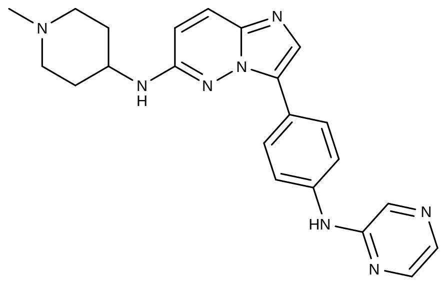	phenyl	0.76	0.61	1.3
4	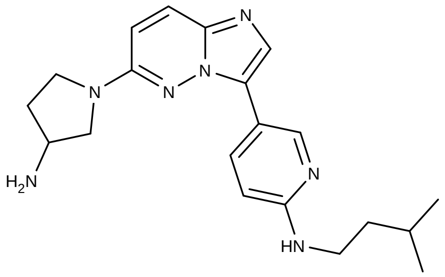	pyridine	0.30	0.22	1.4
5	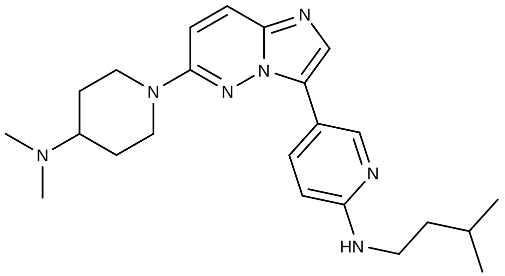	pyridine	0.54	0.39	1.4

6	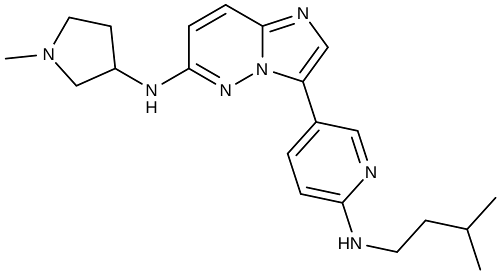	pyridine	0.59	0.42	1.4	
7<br>**Cpd D**	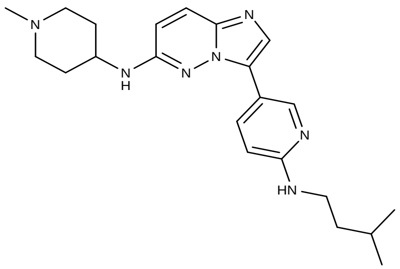	pyridine	0.36(0.08)	0.31(0.10)	1.1	trophozoite (before)
8	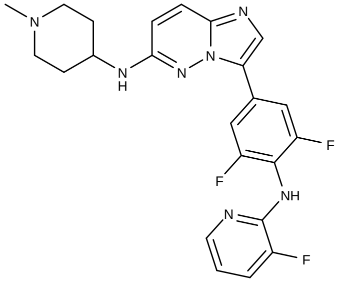	difluorophenyl	0.99	0.66	1.5	
9	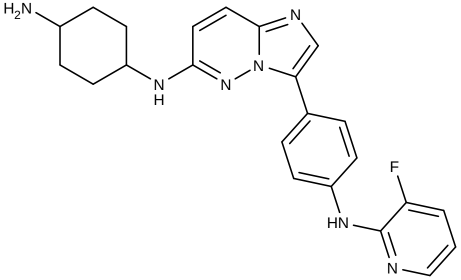	phenyl	0.62	0.40	1.6	

10	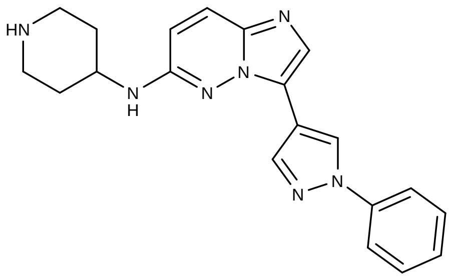	pyrazole	0.72	0.46	1.6
11	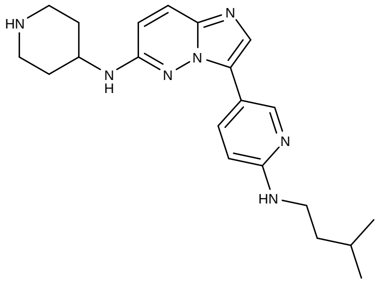	pyridine	0.96	0.60	1.6
12	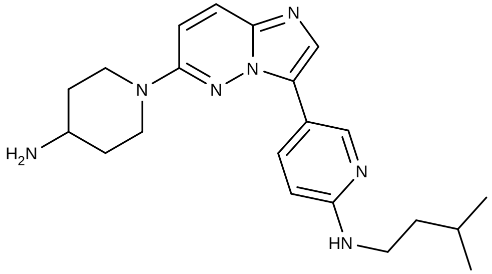	pyridine	0.41	0.25	1.6

13	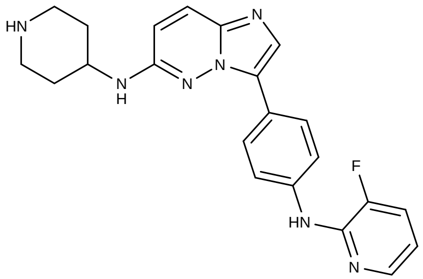	phenyl	1.25	0.76	1.7
14	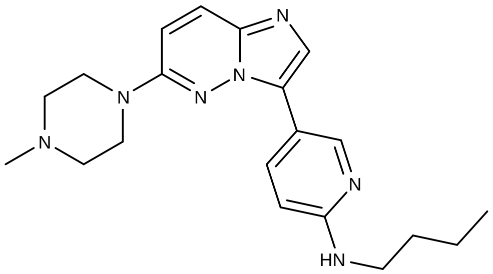	pyridine	2.10	1.18	1.8
15	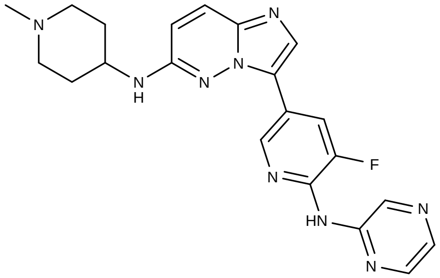	fluoropyridine	1.74	0.96	1.9

16	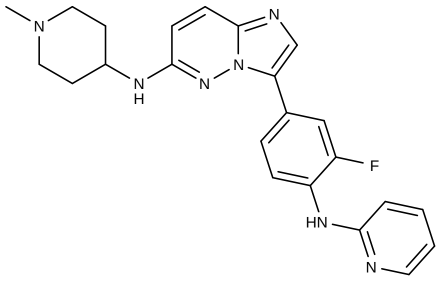	fluorophenyl	0.80	0.40	2.0
17	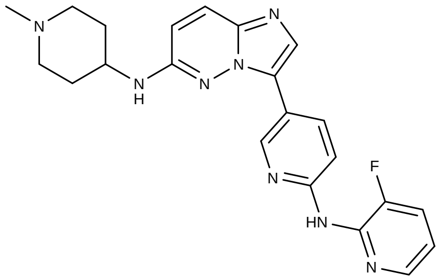	pyridine	1.15	0.58	2.0
18	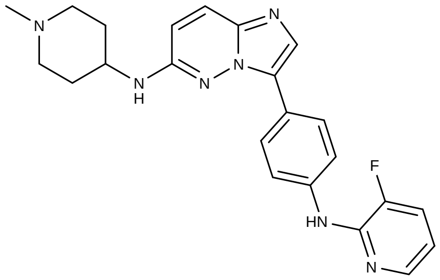	phenyl	1.22	0.60	2.0
19	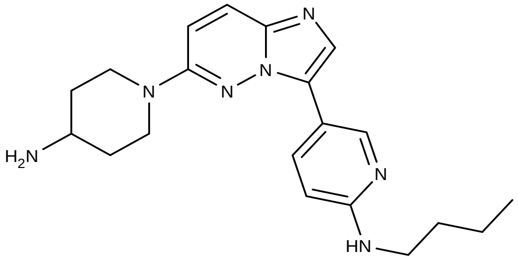	pyridine	0.57	0.26	2.2

20<br>**Cpd. E**	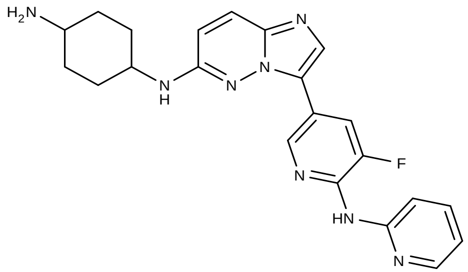	fluoropyridine	0.30(0.07)	0.14(0.02)	2.1	trophozoite<br>(before)
21	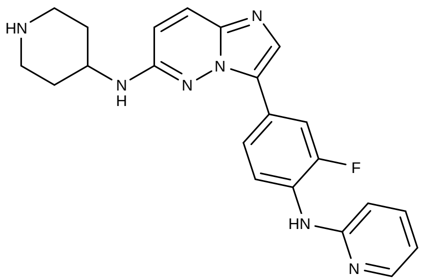	fluorophenyl	1.63	0.69	2.4	
22	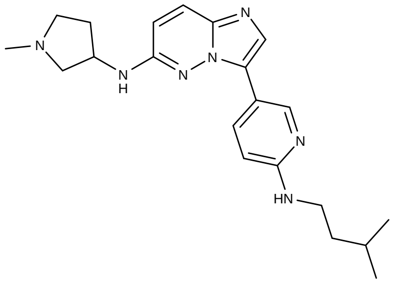	pyridine	0.55(0.08)	0.30(0.14)	1.8	trophozoite<br>(before)
23	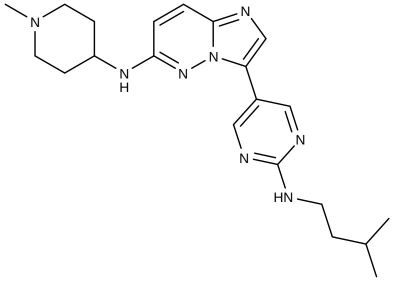	pyrimidine	2.49	0.99	2.5	

24	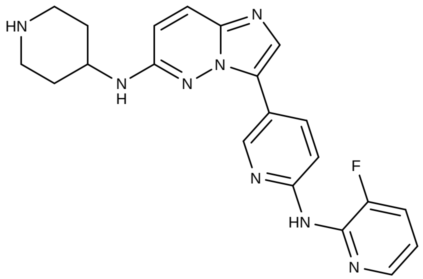	pyridine	2.45	0.71	3.4
25	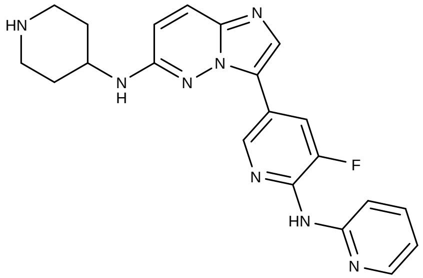	fluoropyridine	1.96	0.54	3.6
26	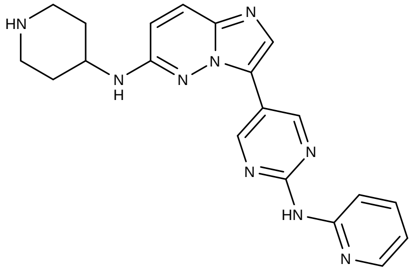	pyrimidine	0.83	0.20	4.2

27	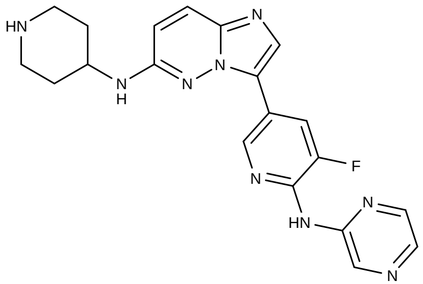	fluoropyridine	4.30	0.84	5.1	
28	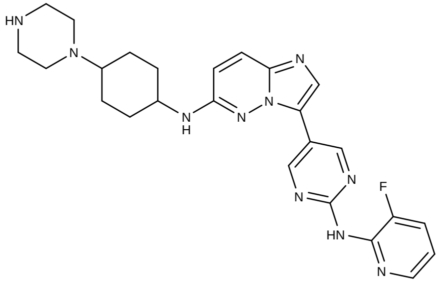	pyrimidine	0.17(0.01)	0.04(0.01)	4.4	late schizont (after)
29	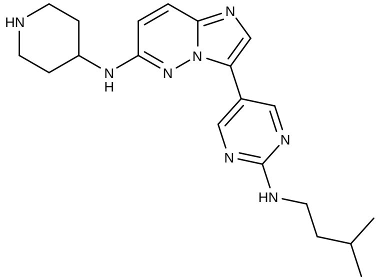	pyrimidine	3.46	0.64	5.4	

30	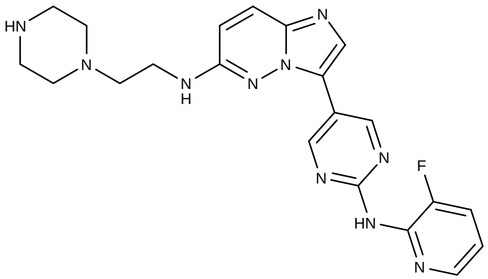	pyrimidine	0.31	0.05	6.0	
31	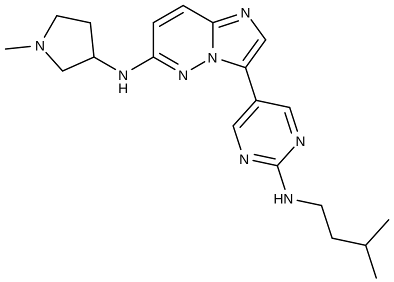	pyrimidine	1.66(0.65)	0.24(0.05)	7.0	late schizont (after)
32	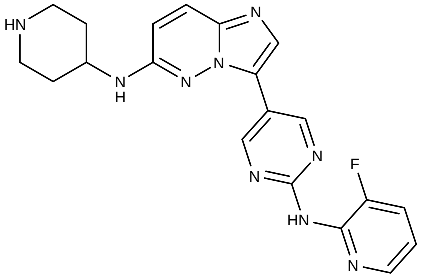	pyrimidine	1.79	0.11	16.6	

33	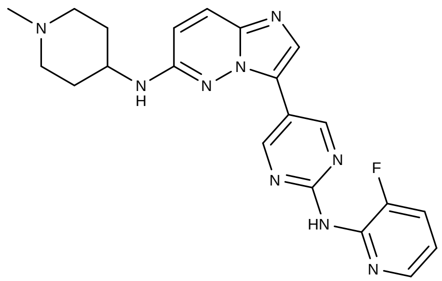	pyrimidine	100	0.03	>1000	
34	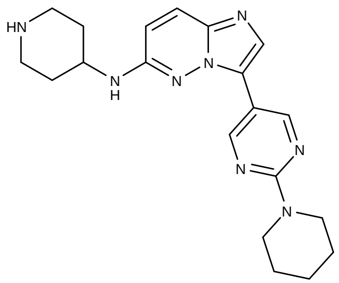	pyrimidine	> 1000	1.18	> 847	
35	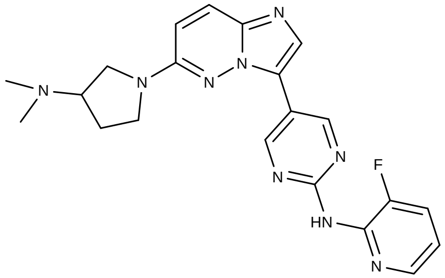	pyrimidine	> 1000	0.81(0.45)	> 1000	late schizont (after)
36 **Cpd A**	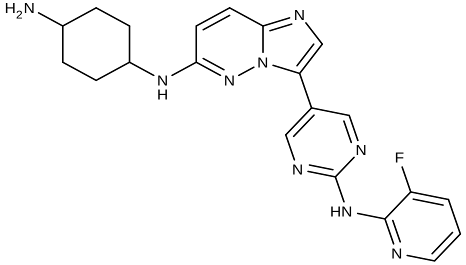	pyrimidine	> 1000	0.02(0.01)	> 1000	late schizont (after)

37	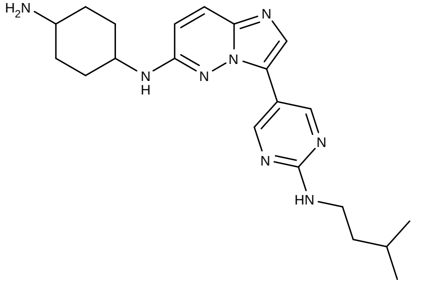	pyrimidine	> 1000	0.65	> 1000	
38	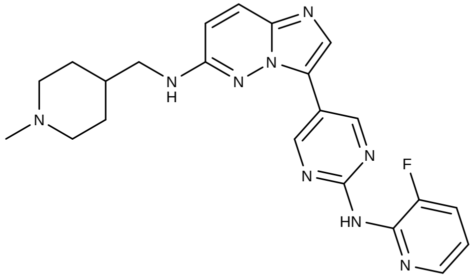	pyrimidine	> 1000	0.05(0.02)	> 1000	late schizont (after)
39 **Cpd. B**	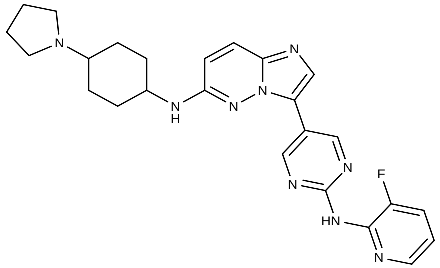	pyrimidine	> 1000	0.02(0.01)	> 1000	late schizont (after)
40	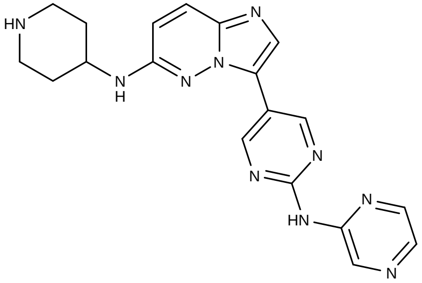	pyrimidine	> 1000	1.23	> 813	

41 **Cpd. C**	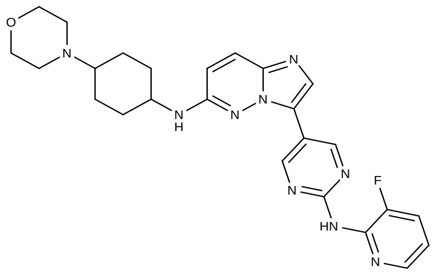	pyrimidine	> 1000	0.04(0.01)	> 1000	late schizont (after)

[a]ring-stage parasites treated with 10x EC50 concentration of compound for 48h followed by microscopic examination of Giemsa-stained parasite smears.

Table S2. Substituting the gatekeeper residue of PKG with a large glutamine residue results in a loss of potency of class 1, pyrimidine-linked compounds. Data shown are from a single experiment other than where the standard deviation from the mean of two independent experiments is indicated in parentheses after the mean EC_{50}.

Compound	Structure	Enzyme inhibition IC_{50} (µM)			Selectivity	Parasite inhibition EC_{50} (µM)		Ratio
		CDPK1	PKG	PKG T618Q	PKG T618Q/ PKG	3D7	3D7 PKG T618Q	3D7PKG T618Q/ 3D7
7 (Cpd D)	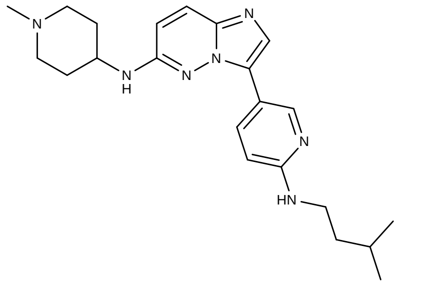	0.013	0.484	15.51	32	0.427 (0.045)	0.477 (0.180)	1
20 (Cpd E)	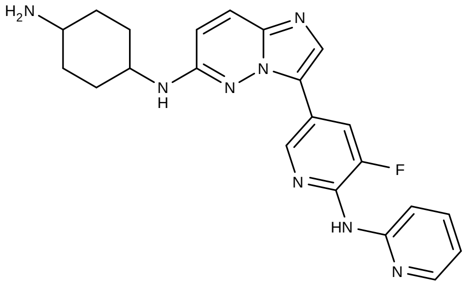	0.008	0.012	15.33	1325	0.210	0.386	2

22	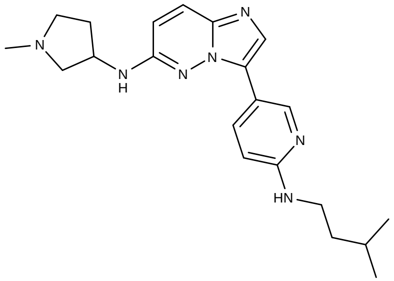	0.074	>1	37.53	<38	0.215	0.303	1
28	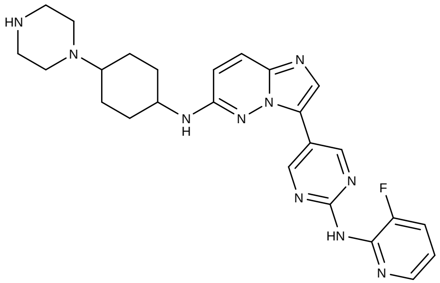	0.012	0.003	31.69	12100	0.059	0.640	11
36 (Cpd A)	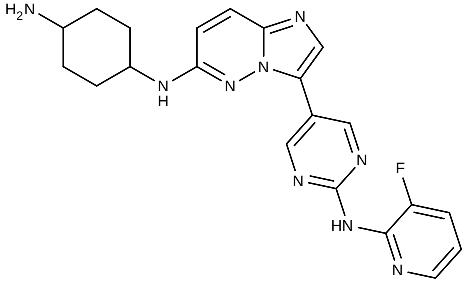	0.008	0.002	10.96	5641	0.034 (0.006)	0.901 (0.483)	27

38	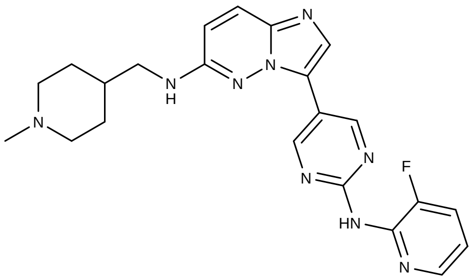	0.008	0.004	43.52	12187	0.073	0.859	12
39 (Cpd B)	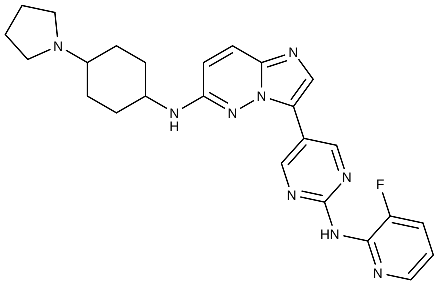	0.009	0.002	25.54	16520	0.034	0.982	29
41 (Cpd C)	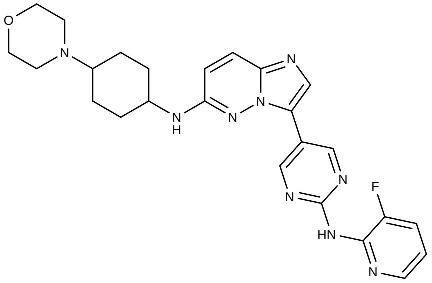	0.011	0.013	12.70	957	0.042 (0.018)	0.923 (0.226)	22

42	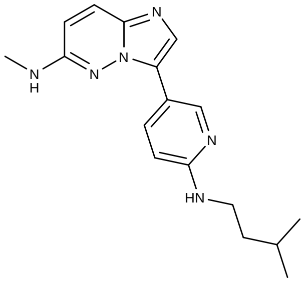	0.081	nd	nd	—	0.836	1.231	1
43	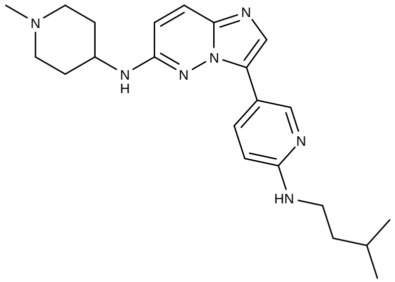	0.088	nd	nd	—	0.079	0.093	1
44	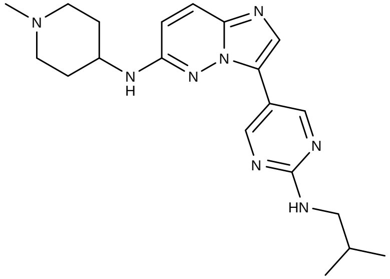	0.065	nd	nd	—	0.236	0.477	2

1	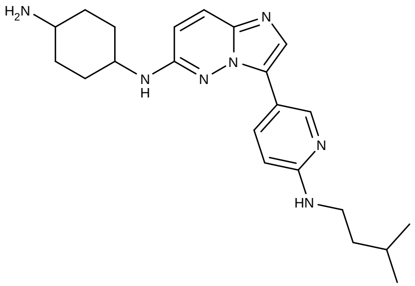	0.010	0.109	6.236	57	
2	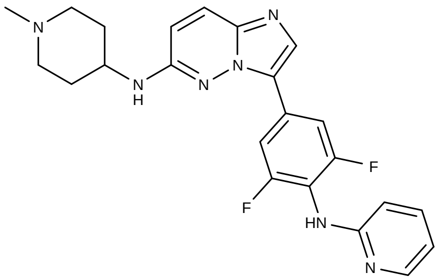	0.026	0.069	12.810	186	
8	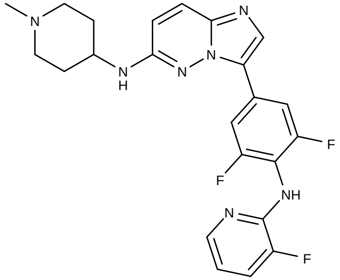	0.017	0.237	64.64	272	

9	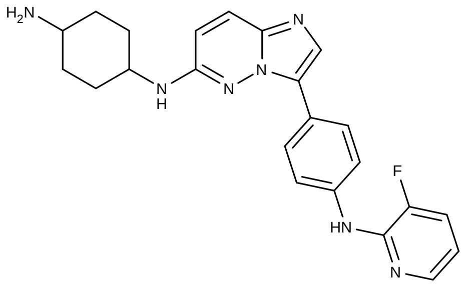	0.034	0.048	16.84	349	
11	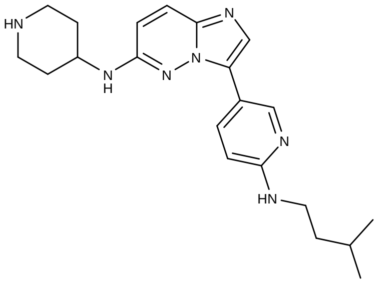	0.015	0.256	256.30	1000	
13	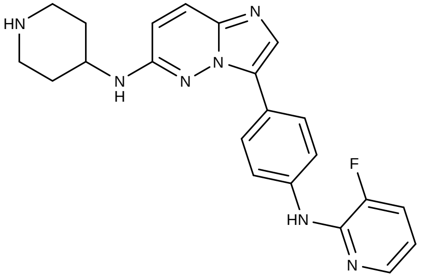	0.014	0.162	3.68	23	

15	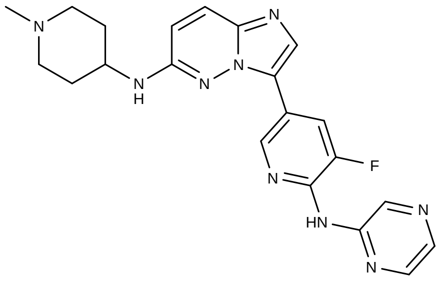	0.020	0.249	51.27	206	
16	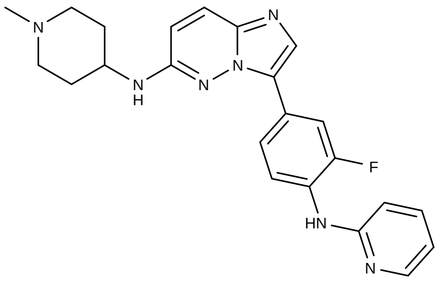	0.011	0.088	10.61	121	
17	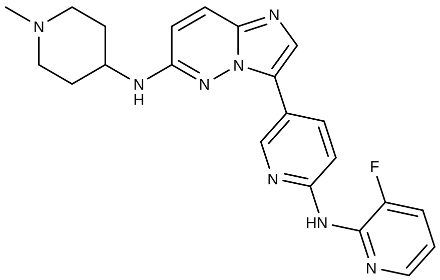	0.009	0.118	12.06	102	

18	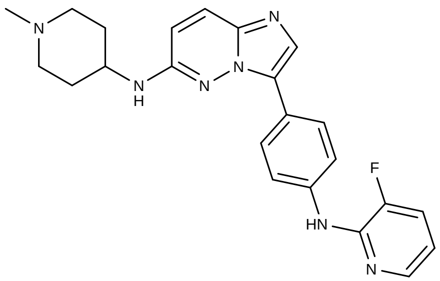	0.016	0.404	15.67	39	
21	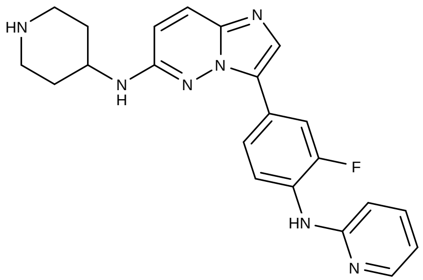	0.010	0.087	3.77	44	
23	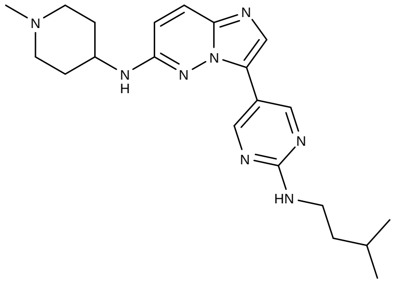	0.014	0.160	4.76	30	

24	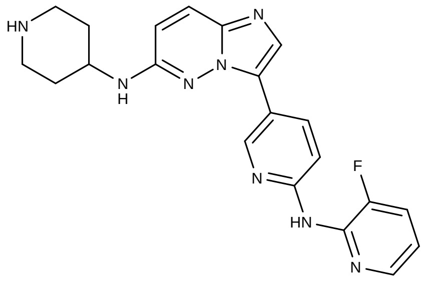	0.011	0.062	1.25	20	
25	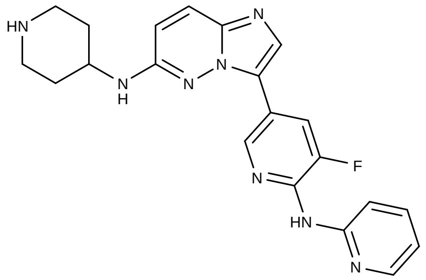	0.013	0.054	9.93	182	
26	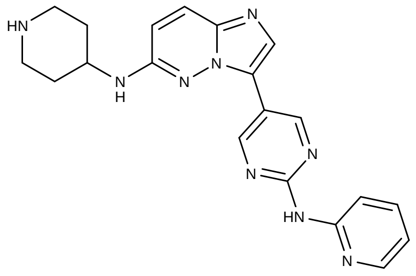	0.009	0.020	3.28	161	

27	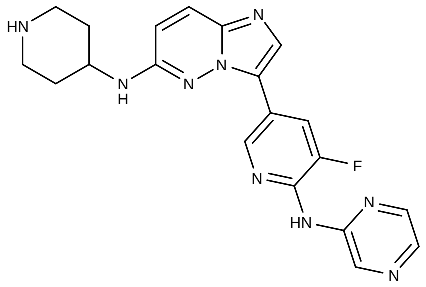	0.017	0.101	10.01	99	
29	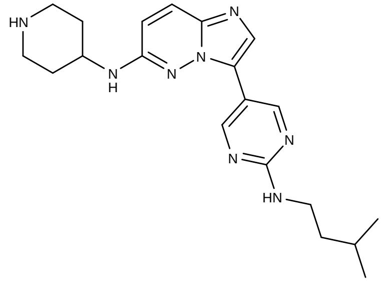	0.018	0.093	0.88	10	
31	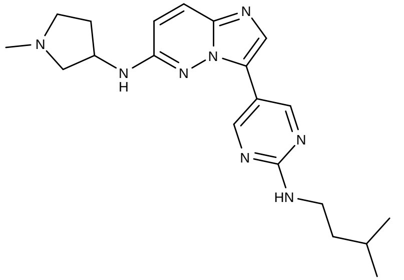	0.093	0.287	5.95	21	

32	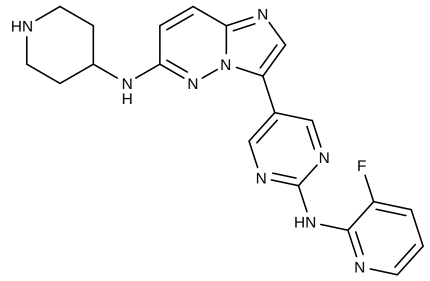	0.010	0.007	6.16	931	
33	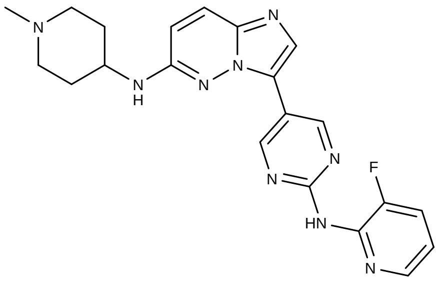	0.013	0.005	10.04	2071	
35	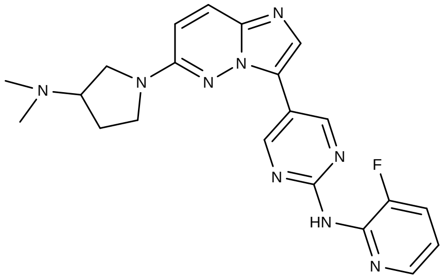	0.017	0.050	528.20	10653	

37	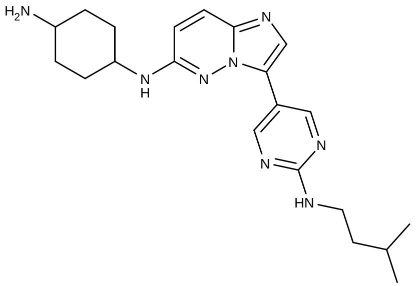	0.016	0.044	2.10	48	
40	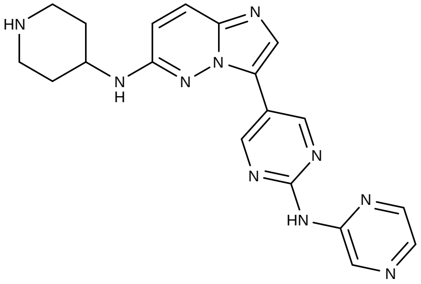	0.014	0.034	4.04	120	

Table S3 Summary of proteins affinity purified with compound D resin, identified by LC-MS/MS.

Accession	Description	MW [kDa]	% Coverage	Unique Peptides	compound A resin		control resin	
**PF3D7_0708400**	heat shock protein 90 (HSP90)	86.1	29.93	24	1.249E6	2.092E6	0.000E0	0.000E0
**PF3D7_1357000**	elongation factor 1-alpha	48.9	10.61	4	4.296E6	1.649E6	0.000E0	0.000E0
**PF3D7_1325100**	phosphoribosylpyrophosphate synthetase	49.4	10.76	4	7.729E5	1.187E6	0.000E0	0.000E0
**PF3D7_1008700**	tubulin beta chain	49.7	5.39	2	3.230E5	4.011E5	0.000E0	0.000E0
